# Supplementary material for: Dynamic bi-directional phosphorylation events associated with the reciprocal regulation of synapses during homeostatic up- and down-scaling
Source: Cell Rep. 2021 Aug 24;36(8):109583. doi: 10.1016/j.celrep.2021.109583 (PMC8411114; doi:10.1016/j.celrep.2021.109583)
Supplement: Document S1. Figures S1–S7 [file mmc1.pdf]

Cell Reports, Volume *36*

Supplemental information

Dynamic bi-directional phosphorylation events associated with the reciprocal regulation of synapses during homeostatic up- and down-scaling

Kristina Desch, Julian D. Langer, and Erin M. Schuman

Supplemental Figures

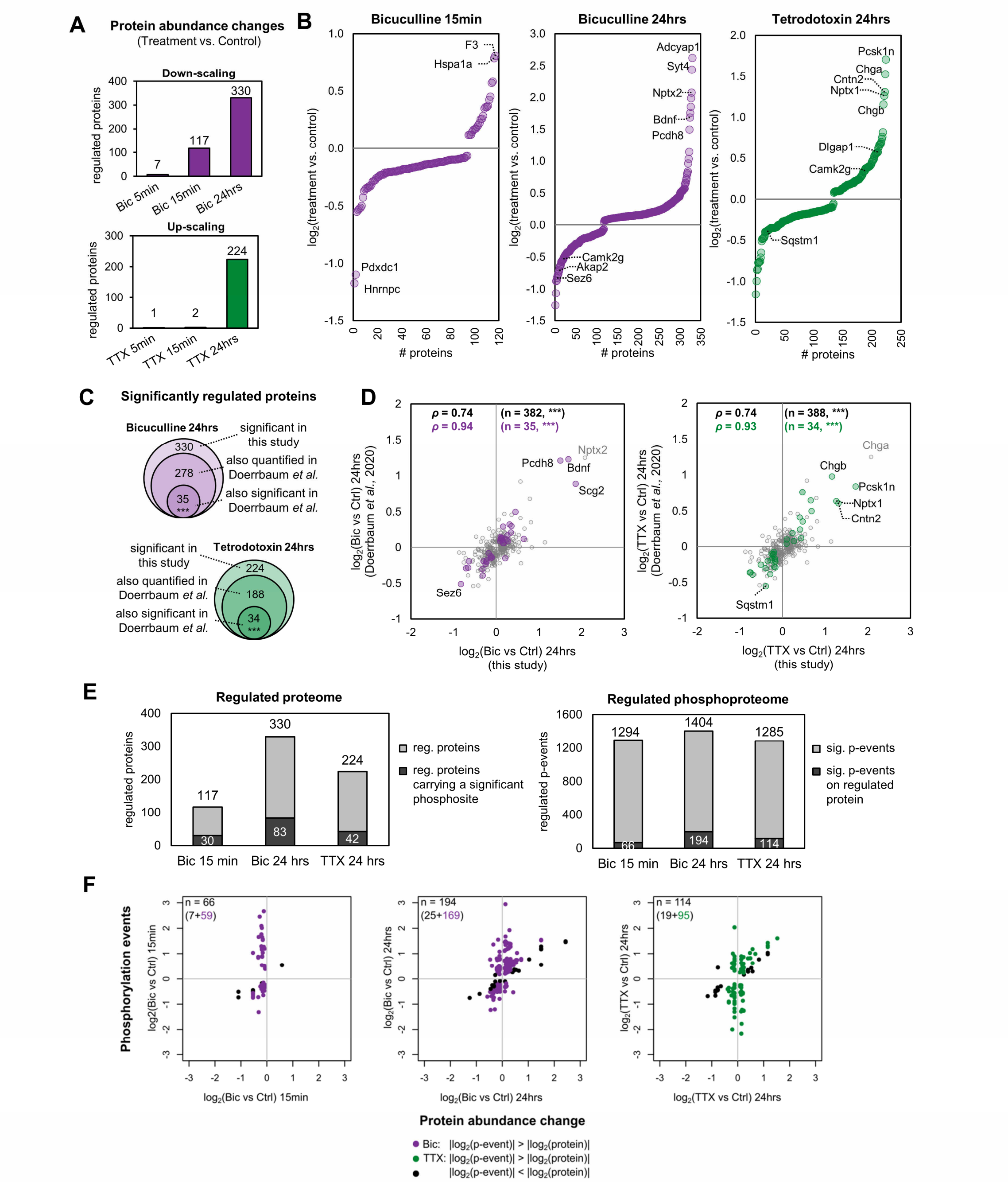

Figure S1 – Remodeling of the cortical proteome during homeostatic synaptic scaling. Related to Figure 1.

(A) Bar chart depicts the number of significantly regulated proteins at each time point of Bic or TTX treatment compared to the control group (Benjamini-Hochberg correction; FDR < 0.01).

(B) Rank-ordered $\log_2$ fold changes of the regulated proteins (treatment vs. control) for the three time points showing the greatest changes in protein abundance: Bic treatment for 15 min and 24 hrs and TTX treatment for 24 hrs.

(C) Comparison of the significantly regulated proteins from this experiment to Doerrbaum *et al.* (see Methods), 278 of the 330 proteins regulated by Bic and 188 of 224 regulated by TTX were also identified in their dataset. Significantly more proteins were regulated in experiments of both studies after 24 hours of Bic and TTX treatment than by chance (Fisher‘s exact test; *** p <0.001; fold enrichment (FE): FE_{Bic} = 3.4, FE_{TTX} = 2.9).

(D) Pearson correlations of the log_2 fold changes (treatment vs. control) of the proteins quantified in both studies were significant (*** $p < 0.001$): The Pearson correlation coefficient (ρ) of the overlapping proteins significantly regulated after 24 hours Bic or TTX treatment in at least one of the studies was 0.74 in both cases ($n_{Bic} = 382$, $n_{TTX} = 388$; grey). The Pearson correlation coefficient of the overlapping proteins exhibiting significant regulation in both studies was 0.94 or 0.93, respectively ($n_{Bic} = 35$, $n_{TTX} = 34$; purple/ green).

(E) Bar plot (left) shows the significantly regulated proteins after Bic treatment for 15 min and 24 hrs or TTX treatment for 24 hrs, highlighting the proportion of regulated proteins that carry at least one significantly regulated phosphorylation site in the same condition in a dark shade. Bar plot (right) displays the significantly regulated phosphorylation events after Bic treatment for 15 min and 24 hrs and TTX treatment for 24 hrs, highlighting the proportion of events that are located on a protein exhibiting significant abundance changes in the same condition.

(F) Scatterplot shows the log_2 fold changes of the overlapping subset of regulated phosphorylation events versus the log_2 fold changes of the corresponding regulated protein. In cases where the change of the phosphorylation event was greater than that of the protein it is located on, the dots are highlighted by color. Black dots indicate a greater change in protein abundance. The majority of phosphorylation events were found to be much more dynamic in all three conditions. The amplitude of regulation on phosphorylation level exceeded those on proteome level. This indicates the activity-dependent changes in phosphopeptide abundance observed in our dataset mostly arise from differential phosphorylation rather than proteome remodeling.

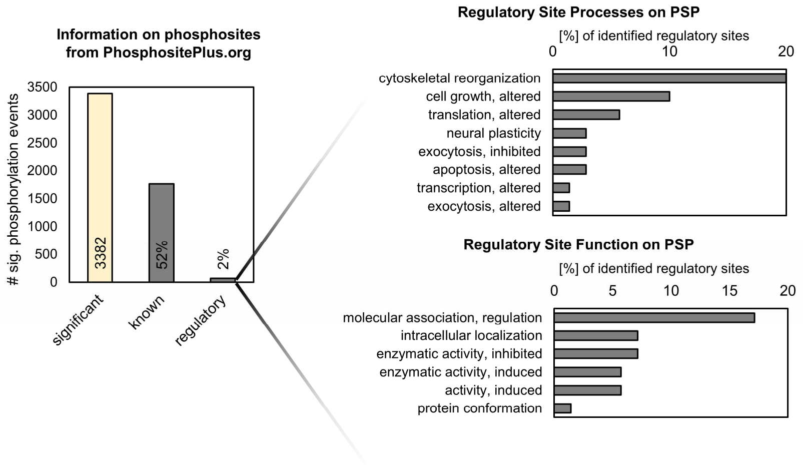

Figure S2 – Annotation of regulated phosphorylation events across all experimental groups. Related to Figure 1.

Significant change in phosphorylation was detected for 3,382 different phosphorylation events regulated along all experimental conditions (Benjamini-Hochberg correction; FDR <0.01). Information on the sites was assigned using the annotations of the PhosphoSitePlus database (Hornbeck et al., 2015). About 50% of the activity-dependent events had been identified in previous experiments (known) and 2% had a regulatory annotation (regulatory). On the left, the terms assigned to characterize the process (top) or function (bottom) of the regulatory sites are listed.

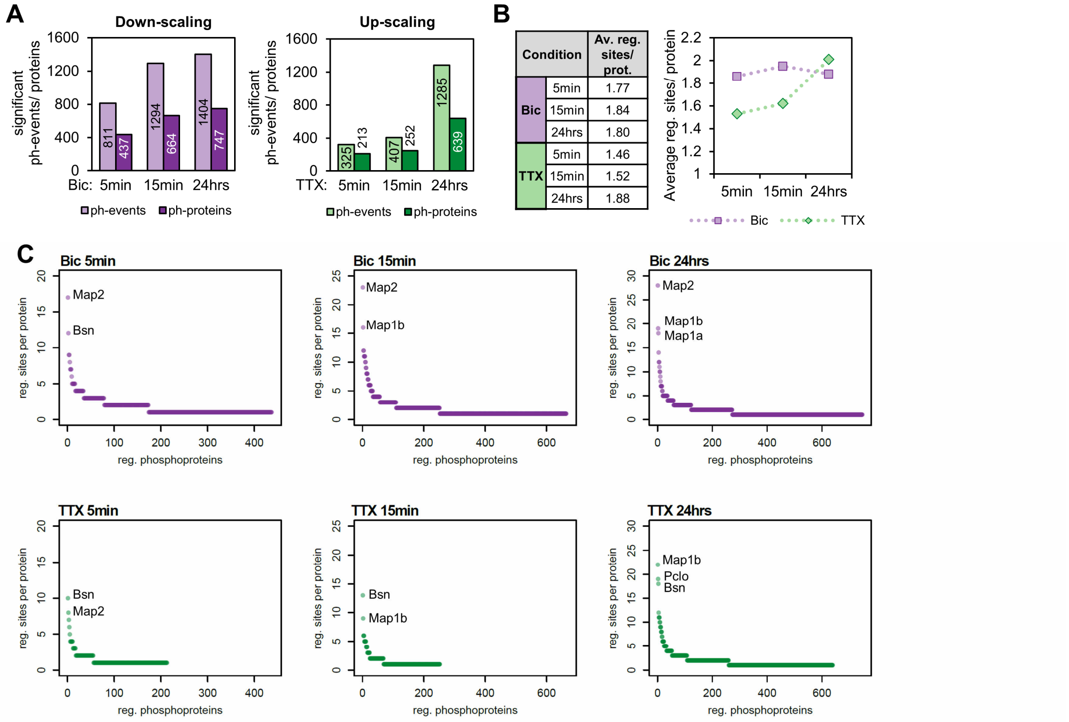

Figure S3 – Activity-sensitive multi-site phosphorylation. Related to Figure 1 and 2.

(A) Temporal distribution of regulated phosphorylation events and unique phosphoproteins. The bar charts represent the number of significantly regulated phospho-events and unique phosphoproteins harboring at least one of these events for each time point of down- (left) or up-scaling (right). Phosphorylation events exhibiting significant regulation comparing treatment versus control condition (Benjamini-Hochberg correction; FDR < 0.01) are shown in a lighter shade, unique phosphoproteins are depicted in a darker shade.

(B) Average phospho-regulation per protein. Table and diagram indicate the average number of phosphorylation sites per protein regulated during 5 min, 15 min or 24 hrs of homeostatic up- or down-scaling (Benjamini-Hochberg correction; FDR < 0.01).

(C) Distribution of regulated phosphorylation sites per protein. Plots show the number of regulated phosphorylation sites per protein during down-scaling (top row) or up-scaling (bottom row) separated by time point. Proteins with the highest number of sites in each condition were labeled according to their gene name.

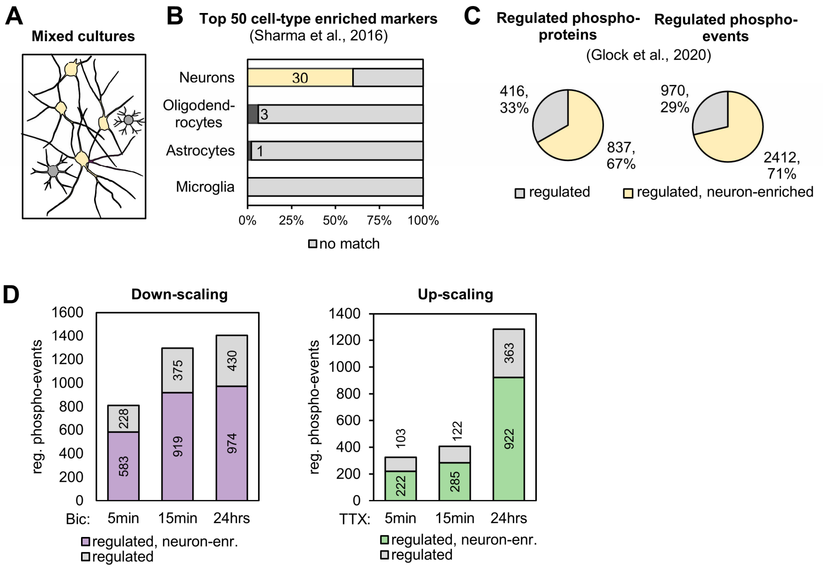

Figure S4 - Excitatory-neuronal contribution to phosphoregulation during homeostatic scaling. Related to Figure 1 and 2.

(A) Scheme representing the mixed primary cortical cell cultures that were used for the phosphoproteomic analysis.

(B) Matching of established, cell-type specific marker proteins to the regulated phosphoproteome. Expression profiles have been previously reported (Sharma et al., 2015) and the top 50 expressed proteins (log_2 fold change compared to all others) for each cell-type were selected and vetted.

(C) Pie chart indicate the proportion of excitatory-neuron specific markers on the level of regulated phosphoproteins (left; 67%) or regulated phospho-events (right; 71%). Markers were matched by gene name. The neuron-enriched fraction is highlighted in yellow. The classifier to bioinformatically filter for transcripts preferentially expressed in excitatory neurons has been previously reported (Glock et al., 2020); for details see Methods.

(D) Bar charts show the number of regulated phosphorylation events per time point during either down-scaling (left) or up-scaling (right) highlighting the proportion of phospho-events on excitatory neuron-enriched proteins (colored; matched by gene name) using the marker genes reported in (C).

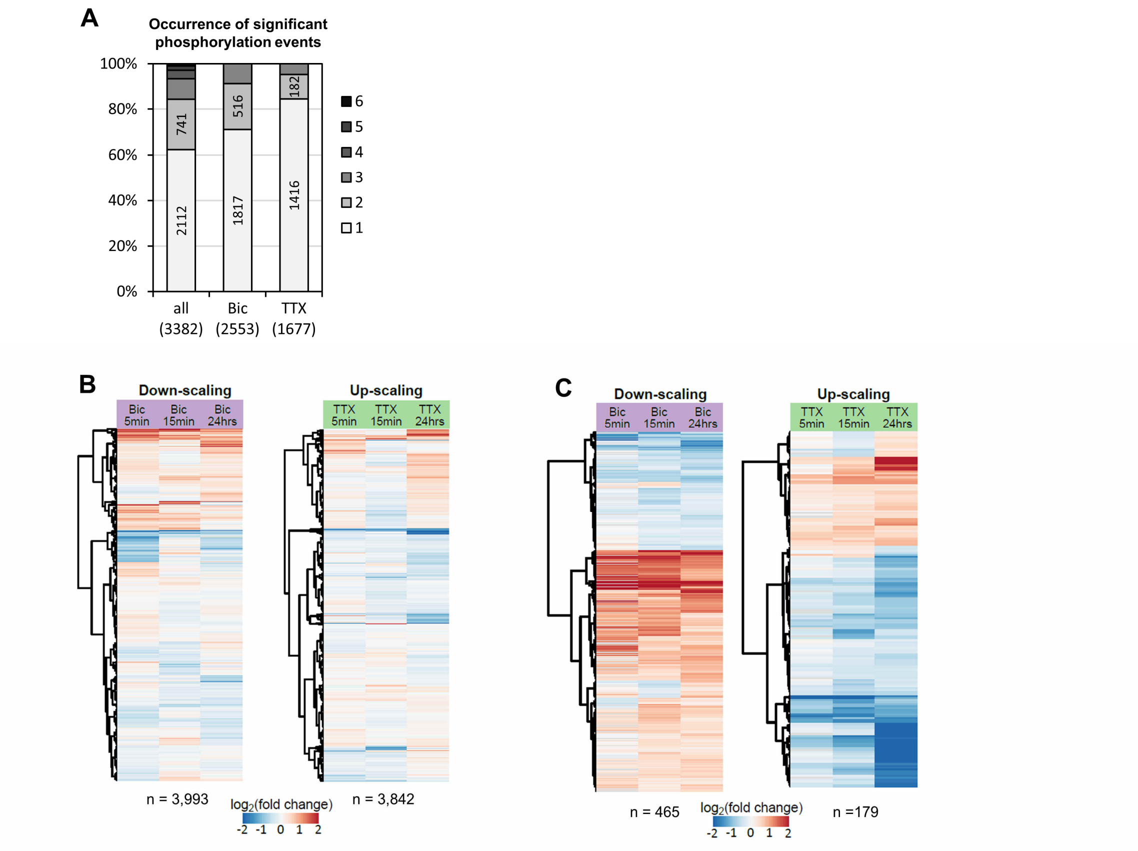

Figure S5 – Significant phosphoregulation across time points. Related to Figure 2 and 3.

(A) Bar chart showing an overview of the occurrence of significantly regulated phosphorylation events across all time points. The first bar summarizes the occurrence of significant regulation of a particular site with respect to all experimental conditions (Bic or TTX: 5 min, 15 min, 24 hrs). 2,112 (62%) of all regulated phosphorylation events were associated with a single time point. The second and third bar summarize the occurrence of significant regulation during up- or down-scaling (n/3). The majority of significant phosphorylation events are regulated in only one of the conditions (down-scaling: 72% and up-scaling 84%)

(B) Temporal profile of the total data landscape of Bic-evoked (left) or TTX-evoked (right) phosphorylation events. Hierarchical clustering (Euclidean distance) was performed on the $\log_2$ fold change of phosphorylation event intensities (treatment vs. control) quantified at each time point of Bic and TTX treatment without applying any filter for significant regulation. The clustering yielded a heatmap with rather diffuse regulation (red: increase in phosphorylation, blue: decrease in phosphorylation), but trending towards persistent regulation which clearly emerged by selection of significant regulators (Figure 2D).

(C) Temporal profile of phosphorylation events significantly regulated at 15 min and 24 hrs (not 5 min) during either Bic- (left) or TTX-stimulation (right). Hierarchical clustering was performed as described in (B). These groups of phospho-events expands the list of bona-fide persistent phospho-events displayed in Figure 2D. Compared to the strictly persistent phospho-events, the number of events in the ‘relaxed’ categories shown here increases ~2.1 or ~2.6 fold for down- or up-scaling, respectively, while largely maintaining their continuous and switch-like regulatory character.

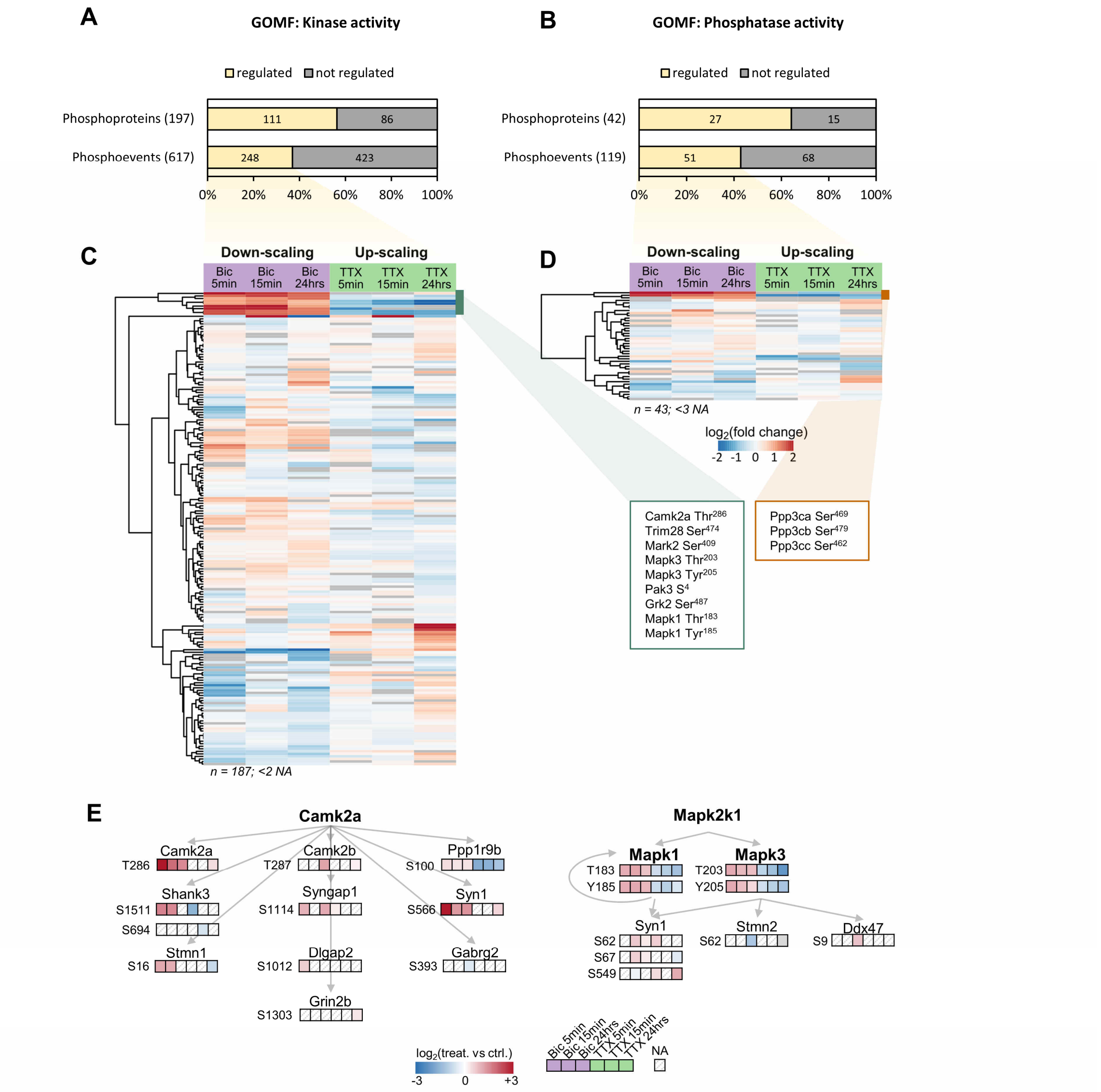

Figure S6 - Phosphoregulated events of kinases and phosphatases. Related to Figure 5.

(A,B) Bar charts indicate the number of all identified kinases (A) or phosphatases (B) on the level of phosphorylated proteins (top row) or phosphorylation events (bottom row). The total number of identifications is indicated in brackets. The proportion of regulated phospho-proteins or -events is highlighted according to significant regulation in at least one time point and condition based on the phosphopeptide-level statistics (FDR < 0.01). Kinases and phosphatases were assigned using the gene ontology annotation for molecular function (GOMF): ‚kinase activity' and ‚phosphatase activity'. We detected phosphoregulation for both, kinases and phosphatases, but identifications and regulatory contribution of phosphatases appeared in a lesser extent compared to the kinases.

(C,D) Heatmap of the $\log_2$-fold changes (intensities of treated vs. control condition) of regulated phosphorylation events that mapped to kinases (C) or phosphatases (D). Phosphorylation events displayed in this heatmaps required a GOMF-term matching kinase or phosphatase activity, significant regulation at at least one condition and were filtered for missing values (NA; grey color) to allow unsupervised clustering (C: < 2 NA; D: < 3 NA). A selection of phospho-sites matching reciprocal clusters were highlighted for both kinases (green box) and phosphatases (orange box).

(E) Phosphorylation status of significantly regulated Camk2a and Mapk1/3 target sites as reported in the rat-specific kinase-substrate database (PhosphoSitePlus) and identified in our dataset. The $\log_2$ fold change of the phosphoregulation (treatment vs. control) is highlighted by color. In case of multiple peptides covering the same phosphorylation site, the $\log_2$ fold change of a particular site was calculated as mean of the events. If the regulation was not significant or if there was no treatment-control pair identified, the box is marked as missing value (NA)

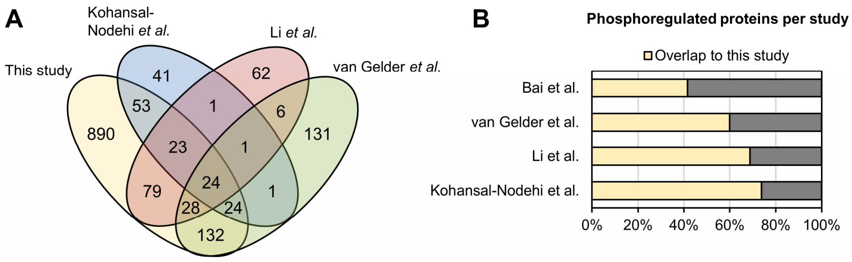

Figure S7 – Comparison of phosphoregulated proteins with other phosphoproteomic studies of synaptic plasticity or neuronal function. Related to Figure 1-5.

(A) Venn diagram highlighting the overlap of proteins that carried at least one significantly regulated phospho-site originating from three different studies focusing on different aspects of synaptic plasticity, such as LTP (Li et al., 2016), depolarization (Kohansal-Nodehi et al., 2016) or mGlur-dependent LTD (van Gelder et al., 2020) with our dataset (for details see Methods).

(B) Barplot indicates the significantly phosphoregulated proteins of each study presented in (A) and including a human AD stage-associated investigation (Bai et al., 2020). Each bar indicates the entity of regulated phosphoproteins per study, the colored fraction highlights the shared phosphoregulated proteins with our dataset (see Methods).
