# Supplementary material for: Dynamic bi-directional phosphorylation events associated with the reciprocal regulation of synapses during homeostatic up- and down-scaling
Source: Cell Rep. 2021 Aug 24;36(8):109583. doi: 10.1016/j.celrep.2021.109583 (PMC8411114; doi:10.1016/j.celrep.2021.109583)
Supplement: Document S2. Article plus supplemental information [file mmc10.pdf]

Cell Reports

Resource

Dynamic bi-directional phosphorylation events associated with the reciprocal regulation of synapses during homeostatic up- and down-scaling

Graphical abstract

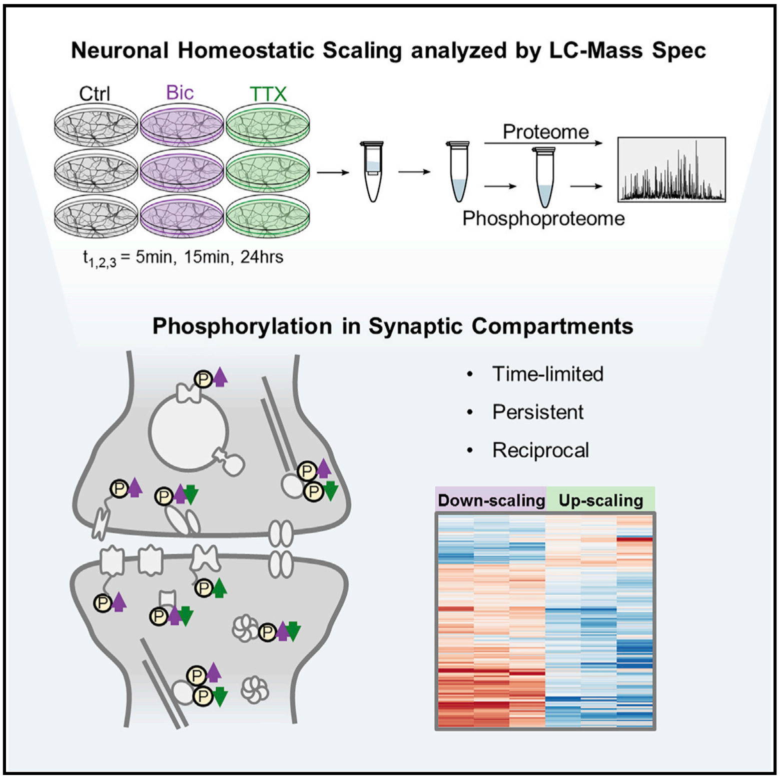

Authors

Kristina Desch, Julian D. Langer, Erin M. Schuman

Correspondence

julian.langer@brain.mpg.de (J.D.L.),
erin.schuman@brain.mpg.de (E.M.S.)

In brief

Using LC-MS/MS, Desch et al. investigate protein phosphorylation as an additional layer of regulation shaping homeostatic synaptic scaling. Characterizing the temporal dynamics of activity-sensitive phosphorylation, they detect potential sensor and/or effector events for up- and down-scaling. They find sustained and reciprocal phosphorylation patterns, which mechanistically couple the opposing scaling phenotypes.

Highlights

- Global proteome and phosphoproteome dynamics following homeostatic synaptic scaling
- Approximately 3,300 activity-sensitive, synapse-associated phospho-events
- Persistent signaling of ~25% of initial phospho-events (min to 24 h)
- Persistent and reciprocal phosphoregulation links synaptic up- and down-scaling

Desch et al., 2021, Cell Reports *36*, 109583
August 24, 2021 © 2021 The Authors.
https://doi.org/10.1016/j.celrep.2021.109583

CellPress

Cell Reports

CellPress
OPEN ACCESS

Resource

Dynamic bi-directional phosphorylation events associated with the reciprocal regulation of synapses during homeostatic up- and down-scaling

Kristina Desch,[1] Julian D. Langer,[1,*] and Erin M. Schuman[1,2,*]
[1]Max Planck Institute for Brain Research, Max von Laue Strasse 4, 60438 Frankfurt, Germany
[2]Lead contact
*Correspondence: julian.langer@brain.mpg.de (J.D.L.), erin.schuman@brain.mpg.de (E.M.S.)
https://doi.org/10.1016/j.celrep.2021.109583

SUMMARY

Homeostatic synaptic scaling allows for bi-directional adjustment of the strength of synaptic connections in response to changes in their input. Protein phosphorylation modulates many neuronal processes, but it has not been studied on a global scale during synaptic scaling. Here, we use liquid chromatography-tandem mass spectrometry (LC-MS/MS) analyses to measure changes in the phosphoproteome in response to up- or down-scaling in cultured cortical neurons over minutes to 24 h. Of ~45,000 phosphorylation events, ~3,300 (associated with 1,285 phosphoproteins) are regulated by homeostatic scaling. Activity-sensitive phosphoproteins are predominantly located at synapses and involved in cytoskeletal reorganization. We identify many early phosphorylation events that could serve as sensors for the activity offset as well as late and/or persistent phosphoregulation that could represent effector mechanisms driving the homeostatic response. Much of the persistent phosphorylation is reciprocally regulated by up- or down-scaling, suggesting that mechanisms underlying these two poles of synaptic regulation make use of a common signaling axis.

INTRODUCTION

Adjustments to neuronal network properties can be achieved by activity-dependent plasticity. Homeostatic synaptic scaling is one such form of plasticity that involves the compensatory and global up- or downregulation of synaptic strength in response to changes in the level of synaptic input. For example, homeostatic up-scaling occurs when network activity is reduced by the cessation of action potentials. In contrast, homeostatic down-scaling can be elicited when network activity is elevated by blocking inhibitory synaptic transmission (Turrigiano, 2008, 2012). Mechanistically speaking, both up-scaling and down-scaling converge on the modulation of synaptic AMPA receptors to bring about the compensatory change in synaptic strength (O'Brien et al., 1998; Turrigiano et al., 1998). Other molecules like NMDA-type glutamate receptors, postsynaptic scaffold proteins such as PSD-95 and Homer, or soluble factors like Bdnf have been implicated in synaptic scaling, as have global changes in the synthesis and degradation of proteins (Dörrbaum et al., 2020; Ehlers, 2003; Schanzenbächer et al., 2016).

For scaling to occur, neurons must possess molecular mechanisms that sense the change in overall activity level (sensors) and then implement the scaling response (effectors). Both modeling and experimental studies suggest that intracellular Ca^{2+} levels may serve as an activity-regulated early signal during scaling (Ibata et al., 2008; Marder and Prinz, 2003; Thiagarajan et al., 2005). Phosphorylation is an important and ubiquitous post-translational modification that is often Ca^{2+} sensitive and can alter a protein's catalytic activity, localization, interactions, or stability (Humphrey et al., 2015). Ca^{2+}-sensitive kinases and phosphatases enable differential phosphorylation and dephosphorylation of various target proteins. For example, Ca^{2+}/calmodulin-dependent protein kinase II α (Camk2a) is a well-studied kinase that is important for many different forms of plasticity. During long-term potentiation (LTP), Camk2a undergoes increases in auto-phosphorylation on Thr^{286}, resulting in Ca^{2+}-independent kinase activity (Fukunaga et al., 1993; Miller and Kennedy, 1986). While phosphorylation events are perhaps ideal for the initial detection of plasticity-induced stimuli, the long-term regulation of kinases (including Camk2a) and phosphatases can also mediate persistent aspects of synaptic and behavioral plasticity (Farley and Schuman, 1991; Malinow et al., 1988; Martin et al., 1997).

Many synaptic receptors and scaffold elements are known to be phosphorylated in the context of normal synaptic function (Greengard et al., 1993; Nestler and Greengard, 1983) and plasticity (Diering et al., 2017; Engholm-Keller et al., 2019; van Gelder et al., 2020; Kohansal-Nodehi et al., 2016; Lee, 2006). Data from a previous broad-scale proteomics analysis revealed kinases and phosphatases as the largest differentially regulated group of newly synthesized proteins during homeostatic scaling (Schanzenbächer et al., 2016). Differential protein phosphorylation during synaptic scaling has also been described by candidate-based approaches (Jang et al., 2015; Sanderson et al., 2018). Recently, Yong et al. (2020) described the phosphorylation of the AMPA receptor subunit 2A (Tyr^{876}) during the late phase of homeostatic up-scaling using phospho-deficient

This is an open access article under the CC BY license (http://creativecommons.org/licenses/by/4.0/).

knock-in mice. Other recent studies have examined phosphorylation during sleep (Brüning et al., 2019; Diering et al., 2017) or in short-term plasticity/stimulation (Engholm-Keller et al., 2019; van Gelder et al., 2020; Kohansal-Nodehi et al., 2016; Li et al., 2016) or have studied its role in a disease-related context (e.g., Alzheimer's disease [AD]; Bai et al., 2020).

Here, we investigated proteome-wide protein phosphorylation following global, bi-directional homeostatic scaling in primary cultured cortical neurons. We made use of a bottom-up, liquid chromatography-mass spectrometry (LC-MS)-based proteomics pipeline that enabled us to identify and quantify unmodified and phosphorylated proteins in a global and unbiased manner. We monitored the activity-sensitive phosphorylation events at the timescale of minutes or 1 day following scaling, establishing a dataset of 3,382 differentially regulated phosphorylation events of 1,285 unique proteins associated with different phases and types of homeostatic scaling. We found distinct regulatory signatures converging on proteins of the synaptic compartment that exhibited persistent, reciprocal, and time-sensitive phosphorylation. A quarter of the initial phosphorylation events detected were persistently regulated in a reciprocal manner, reflecting the opposite polarity of up- and down-scaling.

RESULTS

Activity-dependent protein phosphorylation in cortical neurons

To investigate the dynamics of activity-dependent protein phosphorylation in cortical neurons, we conducted MS-based phosphoproteomics during homeostatic plasticity (Figure 1A). Homeostatic up- or down-scaling was induced by treatment with tetrodotoxin (1 μM; TTX) or bicuculline (20 μM; Bic), respectively. We explored the temporal dynamics of protein phosphorylation during plasticity by examining protein phosphorylation after 5 min, 15 min, or 24 h of stimulation. Temporal snapshots of the proteome and the phosphoproteome were acquired by high-resolution LC-MS/MS analyses using a bottom-up, label-free proteomics approach (see Method details; Tables S1 and S2). Each of the six different experimental conditions was analyzed in four independent biological replicates, which were injected in three technical replicate LC-MS/MS runs.

Across all experimental groups, our analysis detected 4,520 phosphorylated proteins identified in depth with 45,691 phosphopeptide species. These phosphorylation events were filtered for reliable phosphorylation site assignment with a location probability greater than 75% (class I), allowing us to map the modification site to a specific amino acid residue (Figure 1B). In downstream analyses, peptides with insufficient modification site localization (class II/III sites) were discarded. The class I phosphorylation events had a depth of 40,395 peptide species, mapping to 26,642 unique phosphorylation sites. The distribution of phosphorylated serine, threonine, and tyrosine residues was 80.2%, 17.7%, and 2.1%, respectively (Figure 1B), similar to what has been observed in other proteomics experiments (Brüning et al., 2019; Ubersax and Ferrell, 2007). We quantified differential phosphorylation by comparing phosphorylated peptide species in each (up- or down-) scaling group to its associated control sample. Only phosphopeptide species passing stringent criteria were used in downstream analyses (see Method details).

The induction of homeostatic up- or down-scaling led to a large and global regulation of protein phosphorylation (see Table S3). Treatment with Bic (down-scaling induction) for 5 min, 15 min, or 24 h resulted in a significant alteration of 811, 1,294, or 1,404 phosphorylated peptide species, respectively (referred to as "regulated phospho-events"; Figure 1C). Treatment with TTX (up-scaling induction) for 5 min, 15 min, or 24 h resulted in a significant alteration of 325, 407, or 1,285 phosphorylation events, respectively (Figure 1D). The above changes in protein phosphorylation could result from plasticity-induced changes in protein levels, which have been previously documented (Dörrbaum et al., 2020; Schanzenbächer et al., 2016). We assessed the overlap of significantly regulated phospho-events with proteins that underwent significant changes in their abundance (Figures S1A–S1D; Table S4) and found a very small overlap (Figures S1E and S1F; 5%, 14%, and 9% of the regulated phospho-events at 15 min Bic, 24 h Bic, and 24 h TTX treatment were also regulated at the protein level).

The above dataset of 3,382 different activity-regulated phosphorylation events was associated with 1,285 unique proteins (Figures 1C, 1D, and S2). Differential phosphorylation spread to a broader target spectrum as the activity manipulation progressed and often led to multi-site phosphorylation (Figures S3A–S3C). To reveal functional classes of the proteins significantly regulated by phosphorylation, we performed a Gene Ontology (GO) overrepresentation analysis of the regulated phosphoproteins (false discovery rate [FDR] < 0.01). The analysis was conducted separately for up- or downregulated phosphorylated proteins. Consistent with the change in synaptic strength elicited by scaling, we found overrepresentation of the term "synapse" during the whole course of stimulation for both up- and down-scaling, indicating the differential phosphorylation of synaptic proteins (Figure 1E). For both up- and down-scaling, there was also significant overrepresentation of "cytoskeleton organization" as the activity manipulation progressed. During Bic-induced down-scaling, additional terms that were consistently overrepresented were "calmodulin binding" and "postsynaptic density." Altogether, the analysis revealed that during up- and down-scaling, there was rapid and long-lasting phosphoregulation in the synaptic compartment and a progressive re-organization of the cytoskeleton.

As the experiments were performed using mixed cultures obtained from the cerebral cortex, we assessed regulatory contribution of cell-type-specific proteins using the top 50 markers of a previously published cell-type-resolved proteomic dataset (Sharma et al., 2015) or an excitatory neuron-specific classifier (Glock et al., 2020). We mapped the markers to the differentially regulated phosphoproteome (Figures S4A–S4D) and found that neuron-specific phosphoproteins were a dominant contributor (overall 67%; 71% on event level) to the regulation observed during the homeostatic response.

Phosphoprotein dynamics during synaptic scaling

How is protein phosphorylation regulated over time during homeostatic scaling? For both up- and down-scaling, the extent of phosphoregulation clearly increased with the duration of the manipulation (Figures 1C and 1D). While most of the regulated

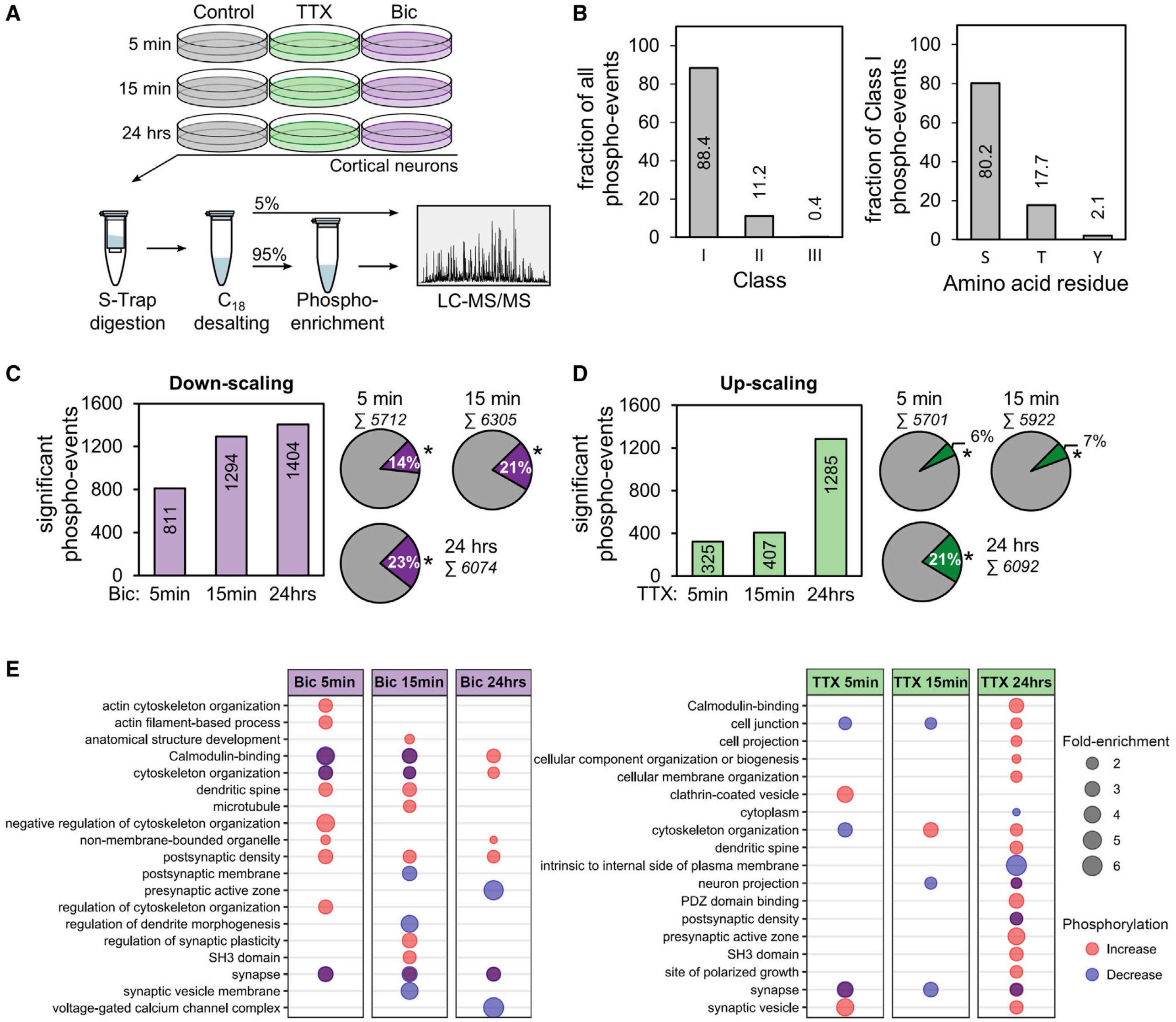

Figure 1. Quantitative LC-MS/MS analysis of activity-dependent phosphorylation in cortical neurons

(A) Illustration of the experimental workflow. Cultured cortical neurons (DIV 19-20) were treated with tetrodotoxin (1 μM; TTX) or bicuculline (20 μM; Bic) for 5 min, 15 min, or 24 h. Subsequently, the cells were harvested and lysed. Digestion was performed using a suspension trapping protocol (S-Trap). After purification of the peptides, one part of the sample (5%) was directly investigated via LC-MS/MS analysis. A second part (95%) was retained for enrichment of phosphorylated peptides using TiO_2-beads and then analyzed via LC-MS/MS (see Method details). For each of the six different conditions, four independent biological replicates were prepared, which were measured in three separate LC-MS/MS runs.

(B) Overview of the identified phosphopeptide species. The left graph shows the distribution of phosphorylated residues of all quantified phosphorylation events according to their localization probability (class I, >75% prob.; class II, 50%–75% prob.; and class III, <50% prob.). The right graph indicates the distribution of phosphorylated amino acid residues of all class I phosphorylation events.

(C and D) Temporal profile of phosphorylation events. The bar charts show the number of significantly regulated phosphorylation events (Benjamini-Hochberg correction; FDR < 0.01) comparing Bic (C) or TTX (D) treatment versus the control at each time point. The pie charts indicate the proportion of significantly (*) and not significantly regulated phosphorylation events.

(E) Gene ontology (GO) overrepresentation analysis of phosphorylated proteins for all three time points of Bic (left) and TTX (right) treatment (Benjamini-Hochberg correction; FDR < 0.01). Proteins with a regulated phosphorylation site were divided with respect to the nature of the regulation (increase or decrease) prior to GO analysis. The degree of regulation is highlighted by the color, and the fold enrichment is indicated by the size of the dots. If a term was enriched for both increased and decreased phosphorylation, the corresponding dot is shown in overlap and hence appears purple.

phosphorylation events (62%, n = 2,112) were associated with a single time point (down-scaling, 72%; up-scaling, 84%; Figure S5A), there were also a large number of persistent phosphorylation events (observed at all times) for each form of scaling (Figures 2A and 2B). To examine the regulation associated with the different phases of plasticity, we first binned the time-limited

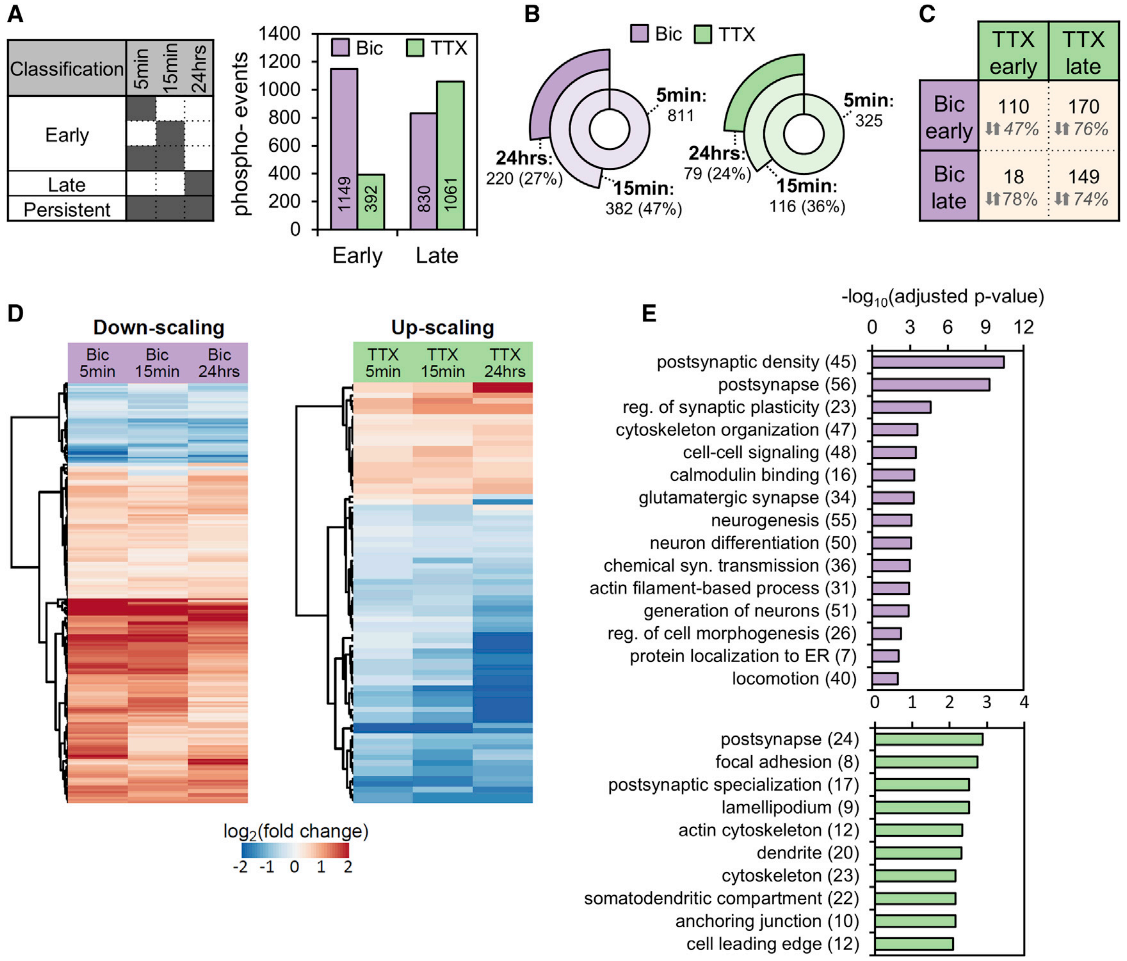

Figure 2. Phosphorylation dynamics during synaptic scaling

(A) Time-limited phosphorylation events were grouped according to the classification indicated in the table (left). Dark color indicates significant regulation at the highlighted time point. The bar chart (right) displays the distribution and number (insets on bars) of early and late phosphorylation events for Bic and TTX treatment.

(B) Significantly regulated phosphorylation events over time. About a quarter (Bic: 27%; TTX: 24%) of the initially regulated phosphorylation events exhibited significant changes at all time points.

(C) Overlap of the phosphorylation events in the temporal groups as observed between the treatment groups is indicated in absolute numbers in the table. The percentage of overlapping phosphorylation events exhibiting reciprocal regulation is written below.

(D) Hierarchical clustering (Euclidean distance) was performed on the $\log_2$-fold changes (treatment versus control) of the phosphorylation event intensities regulated during up- and down-scaling as described in (C). Shown is a heatmap (red, increase in phosphorylation; blue, decrease in phosphorylation) indicating two major clusters for both up- and down-scaling. There was a significant decrease in intensity over time in the down-scaling phosphorylation increase cluster and a significant increase in intensity over time in the up-scaling phosphorylation decrease cluster ($p < 0.05$; ANOVA and Tukey honestly significant difference [HSD] post hoc).

(E) GO overrepresentation analysis of the persistent Bic-evoked (top) and TTX-evoked (bottom) subset of phosphorylation events (Benjamini-Hochberg correction; FDR < 0.01). Selected terms and their adjusted p values are shown (see Table S6 for all terms).

phospho-events for each type of scaling (Figure 2A; Table S5). A large number of temporally specific phospho-events were detected in the early (n = 1,149) and late phases (n = 830) of down-scaling, whereas during up-scaling, the number of phosphorylation events increased dramatically over time (n = 392 and 1,061 for early and late time points, respectively). Most of the time-limited events were specific for either up- or down-scaling. These phosphorylation events could represent sensors/effectors that were specific to the sign of the plasticity. On the other hand, we also observed overlap in the phosphorylation targets observed in up- and down-scaling: 110 events during the early phase and 149 during the late phase of plasticity (Figure 2C). A large fraction of all overlapping regulated phosphorylation events was reciprocal in nature: positively regulated (increased) phosphorylation for one type of scaling and negatively regulated (decreased) in the other. These reciprocally regulated phosphorylation events could represent signaling pathways responsible for the detection of activity offsets from a set point; the sign of the activity offset (increased or decreased) could then be represented by an increase or decrease in phosphorylation.

To investigate the temporal evolution of phosphorylation during homeostatic scaling, we performed a hierarchical clustering analysis of the persistent phosphorylation events. Approximately a quarter of initial phosphorylation events (regulated at 5 min) exhibited significant regulation at all three tested time points (Figure 2B). We subjected the log_2-transformed fold changes of all the persistently regulated phosphorylation events to hierarchical clustering (Figures 2D, S5B, and S5C). The long-lasting phosphorylation events formed two distinct clusters during up- or down-scaling: one cluster represented a persistent increase in phosphorylation, and the other represented a persistent decrease in phosphorylation. During down-scaling, the phosphorylation increase cluster was large, and the phosphorylation decrease cluster was smaller, whereas the opposite was true for up-scaling. This continuous character of the persistent phospho-events was largely preserved when we expanded the bona fide, persistent group to phospho-events that were significantly regulated at 15 min and 24 h (and not 5 min) during up- or down-scaling (Figure S5C).

Which phosphorylated proteins are part of these persistently regulated clusters? We performed GO overrepresentation analyses for the persistently regulated phosphoproteins and found, again, many protein groups related to synaptic function (Figure 2E). For example, for both up- and down-scaling, the term "postsynapse" was overrepresented. Similarly, "postsynaptic density," "regulation of synaptic plasticity," and "chemical synaptic transmission" were overrepresented in the Bic-induced persistent subset of signals and "postsynaptic specialization" in the TTX-induced persistent subset. The term "calmodulin binding" for phosphoproteins regulated during down-scaling was also observed. Indeed, we detected the persistent autophosphorylation site Thr^{286} on Camk2a, which leads to Ca^{2+}-independent enzyme activity (Miller and Kennedy, 1986). We also observed a persistent increase in the phosphorylation of Ser^{421} of Methyl-CpG-binding protein 2 (Mecp2), the Rett syndrome protein implicated in synaptic plasticity and an established Camk2a substrate (Amir et al., 1999; Zhou et al., 2006). Furthermore, phosphorylation at Ser^{556} on Synapsin 1 (Syn1), another Camk2a-regulated site (Czernik et al., 1987), also showed persistent regulation. Both lists of significantly overrepresented terms also included the cytoskeleton: "cytoskeleton organization" and "locomotion" during down-scaling or "actin cytoskeleton" and "cytoskeleton" during up-scaling.

Comparing the phosphorylation pattern of up- and down-scaling

Homeostatic up- and down-scaling involve the reciprocal regulation of synaptic strength in response to activity that is either decreased or increased relative to a set point. Although both forms of scaling converge on the same phenotypic endpoint (the number of synaptically localized AMPA receptors), the cell-signaling mechanisms are not well understood. To examine whether the same proteins were regulated by up- and down-scaling, we expanded the clustering analysis to phosphorylation events that were regulated in at least four of six possible conditions across both experimental groups. The phosphorylation events that were regulated in both scaling groups showed a remarkable reciprocity in the sign of regulation (Figure 3A): upregulated phosphorylation events during down-scaling were downregulated during up-scaling, and vice versa. We generated protein-protein-interaction maps of the reciprocally regulated phosphoproteins of the two major clusters and annotated them (Figure 3B). The smaller cluster of reciprocal phosphorylation events was downregulated during down-scaling and upregulated during the up-scaling. Three small interaction networks were formed by presynaptic proteins (Aak1, Bin1, Amph, or Snap91), proteins associated with ubiquitin signaling (Nedd4l or Lmo7), but also by cytoskeletal proteins (Epb41l3 or Epb41l1). The second cluster of reciprocal phosphorylation events was upregulated during down-scaling and downregulated during up-scaling. In total, nine interaction networks were generated from this set. The smaller subnetworks comprise phosphoproteins directly involved in ubiquitin signaling (Uba1, Uba5, Ube2o, or Herc1) or associated with translation initiation (Eif4b and Eif4g). Another subnetwork contained proteins of the presynaptic compartment following the same regulation pattern. The largest subnetwork comprised phosphoproteins of the postsynaptic compartment. Postsynaptic density protein 95 (Dlg4, PSD-95), a post-synaptic scaffold protein implicated in synaptic plasticity (Bats et al., 2007; Vallejo et al., 2017), emerged as a central hub. Other phosphoproteins that exhibited this reciprocal regulation profile were important cytoskeleton interactors such as Syngap1 or Map1a, but also proteins known to interact with or traffic neurotransmitter receptors (Grip1, Sh3kbp1, or Lrrc7). Of note, phosphorylation sites on some proteins in this network have been reported to affect the protein's function directly (e.g., phosphorylated Ser^{2798}/ Ser^{2804} of the Ryanodine receptor 2 [Ryr2] increases channel conductance) (Ferrero et al., 2007; Huke and Bers, 2008).

To understand how this bi-directional phosphorylation pattern is achieved, we investigated kinases and phosphatases that were differentially phosphorylated by scaling. We detected many regulated phospho-events on kinases and, to a lesser extent, also on phosphatases (Figures S6A–S6D). Prominent activity-dependent kinases were central nodes in the postsynaptic subnetwork. For example, the activating phosphorylation sites of Camk2a (Thr^{286}), ERK1 (Thr^{203}/Tyr^{205}), or ERK2 (Thr^{183}/Tyr^{185}) showed significant reciprocal regulation. Subunits of Ca^{2+}-sensitive phosphatases (Pppc3a-c) were also reciprocally phosphorylated (Figure S6D), though lacking site-specific information to assess functional contribution. We focused the analysis on kinases and next performed kinase-substrate-enrichment analysis (KSEA) where kinase activity (kinase Z score; Figure 3C; Table S7) was calculated from the phosphorylation status of reported substrates (Figure S6E). There, persistent and bi-directional behavior could be detected by the kinase Z scores of Camk2a and upstream of ERK1/2 (Map2k1).

We validated the patterns of Camk2a and MAP-kinase phosphoregulation using protein-specific antibodies together with phospho-specific antibodies (Figure 4). Overall, the pattern of phosphoregulation observed with phospho-specific antibodies was very similar to that observed in the MS data. Using immunoblotting, we found that down-scaling resulted in a significant increase in Camk2a Thr^{286} phosphorylation at both 5 min and 24 h, whereas up-scaling resulted in a trend for enhanced phosphorylation at 24 h (Figures 4A and 4B). For both ERK1 Thr^{203}/

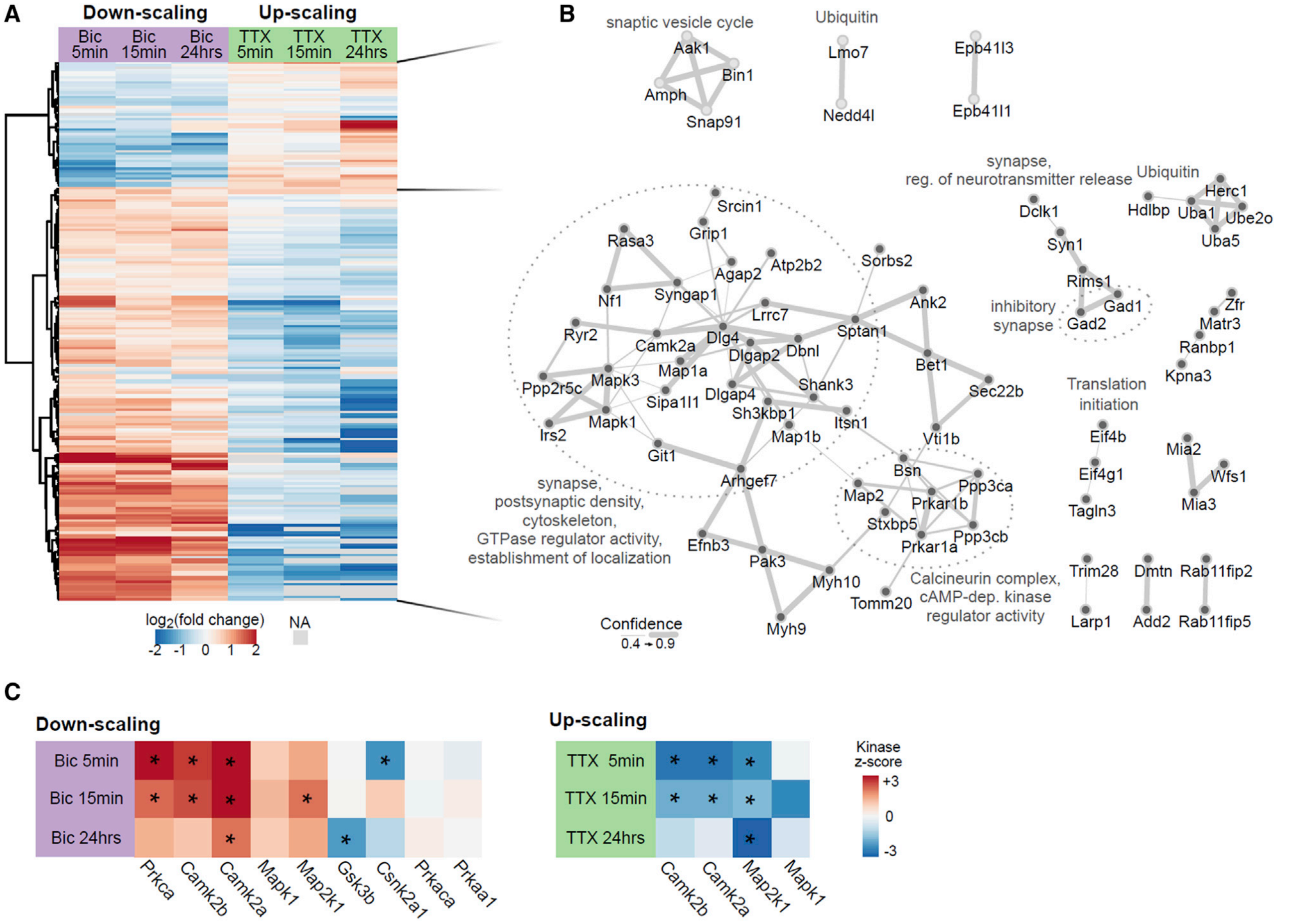

Figure 3. Persistent and reciprocally regulated phosphorylation patterns during up- and down-scaling

(A) Hierarchical clustering analysis shown with a heatmap combining both scaling experiments: the $\log_2$-fold change (treatment versus control) of phosphorylation events that were significantly regulated in at least four of six experimental conditions (n = 219) were clustered (Euclidean distance). Red, increase in phosphorylation; blue, decrease in phosphorylation. Missing values (NA), gray color.

(B) Phosphoproteins of the two major clusters of the heatmap in (A) were analyzed for protein interactions using their gene identifiers and the String database. Cluster membership is indicated by the color of the nodes (light gray nodes, top cluster; gray nodes, bottom cluster). The sub-networks are described by overrepresented GO or manually curated terms (UniProtKB). Width of the connections shows the confidence of the interaction (combined score) as derived from the String database. Unconnected phosphoproteins are not shown.

(C) Prediction of kinase activity was performed via kinase substrate enrichment analysis (KSEA) on the significantly regulated phosphorylation events of each condition (see Method details). Kinase activity is visualized by color according to the kinase *Z* score (red, increase in kinase activity; blue, decrease in kinase activity). Significant regulation is highlighted by an asterisk within the tile of the heatmap (*p < 0.05, FDR < 0.1).

Tyr^{205} and ERK2 Thr^{183}/Tyr^{185}, down-scaling resulted in a significant increase in phosphorylation at both 5 min and 24 h (Figures 4C and 4D). In contrast, up-scaling resulted in a significant decrease in phosphorylation, which was a clear trend at 5 min and significantly different from control at 24 h (Figures 4C and 4D). Taken together, these data suggest that the activity of Ca^{2+}-sensitive kinase Camk2a and ERK1/2 drives persistent phosphorylation during Bic-induced down-scaling, while they appear deactivated during TTX-induced up-scaling.

Mapping the regulation of the synaptic phosphoproteome over time

To examine the different phospho-patterns in the synaptic compartment, we generated time-resolved and site-specific maps of the synaptic phosphoproteome during homeostatic up- and down-scaling (Figure 5; Table S8). The protein-based visualization of phosphoregulation affirmed the similarity between both scaling polarities, as seen by the common phosphoproteins (n = 94, marked with asterisks). Site-specific annotation revealed that different modes of phosphorylation converged on phosphoproteins in both the pre- and postsynaptic compartments. Many phosphoproteins were phosphorylated at more than one phospho-site during the experiment. An example is the differential phosphorylation of Dlg4. During Bic-induced down-scaling, Dlg4 exhibited upregulation at three different sites (Tyr^{240}, Ser^{418}, and Ser^{422}) assigned to all three temporal classes (early, late, and persistent/reciprocal). During TTX-induced up-scaling, significant changes were limited to

CellPress
OPEN ACCESS

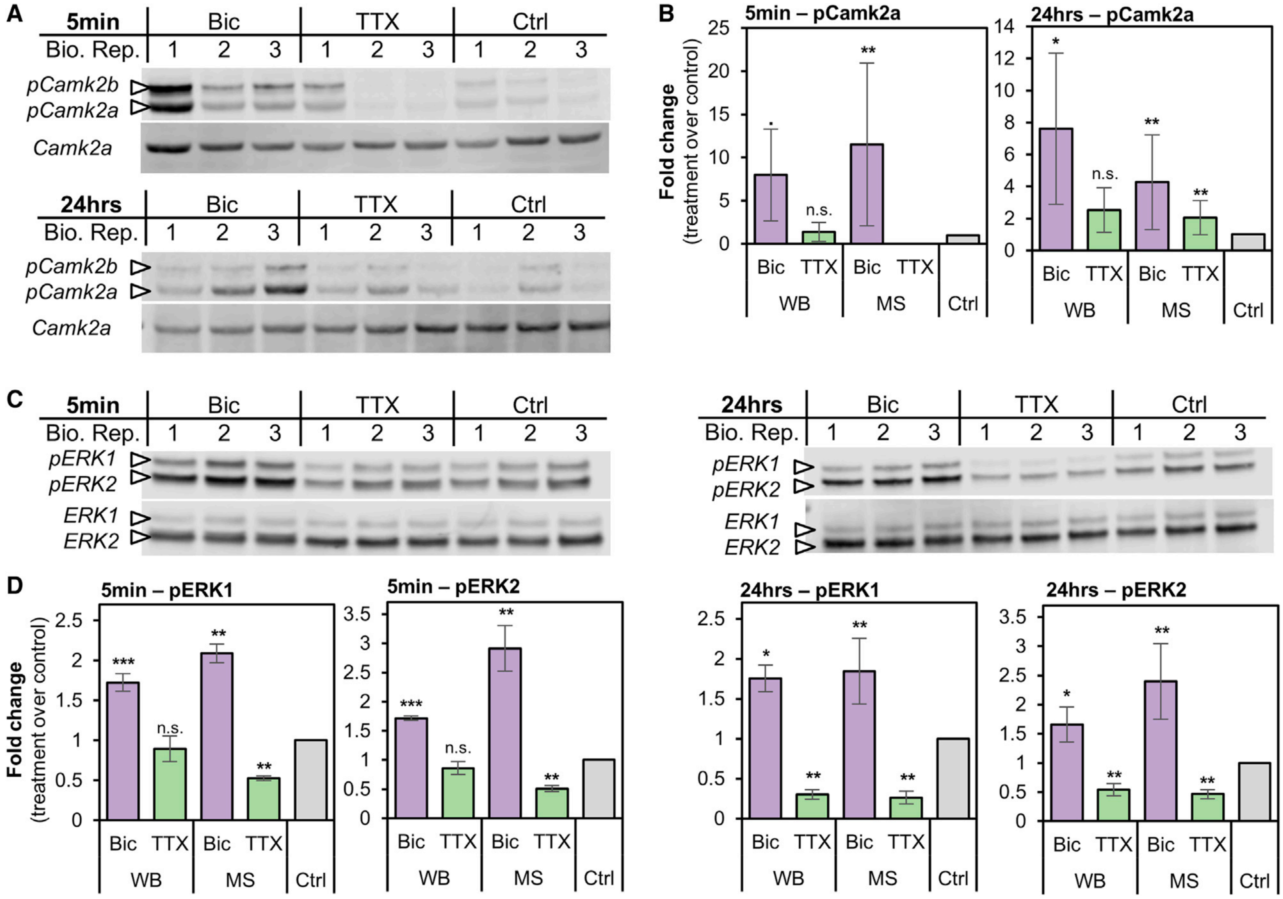

Figure 4. Verification of phosphoregulated Camk2a, ERK1, and ERK2 by immunoblotting

(A) Western blot (WB) images for pCamk2a (pThr^{286}) and total Camk2a. Analyses were performed using three independent biological replicates of Bic-treated, TTX-treated, and untreated (control) neurons for 5 min (top) or 24 h (bottom).

(B) Bar graphs show quantification of pCamk2a detected by WB in comparison to the phosphopeptide's signals quantified via LC-MS/MS (MS) after 5 min (left) or 24 h (right) Bic or TTX stimulation. Protein signals of the blot were quantified based on the intensity ratio of the phosphorylated protein over total protein normalizing treated conditions to the untreated control. Log_2-scaled MS intensity of the phosphorylated peptide is depicted as the fold change of treatment over control as well (see Method details). Error bars represent the standard deviation between biological replicates (WB, n = 3; MS, n = 4). For statistical analysis of the WB data, a two-sided t test was performed: ***$p > 0.001$; **$p < 0.01$; *$p < 0.05$; . $p < 0.1$; n.s., not significant. For MS-data, ** FDR < 0.01.

(C) WB images for pERK1 (pThr^{203}/pTyr^{205}) or pERK2 (pThr^{183}/pTyr^{185}) and total ERK1 and ERK2. Analyses were performed using three independent biological replicates of Bic-treated, TTX-treated, and untreated (control) neurons for 5 min (left) or 24 h (right).

(D) Bar graphs show quantification of pERK1 and pERK2 detected by WB in comparison to the phosphopeptides quantified via LC-MS/MS (MS) after 5 min (left) or 24 h (right) Bic or TTX stimulation. WB and statistical analyses were performed and visualized as described in (B).

three different phospho-sites that were either continuously regulated (Ser^{422}: downregulated) or regulated during the late phase of stimulation (Ser^{73}: upregulated; Ser^{418}: downregulated). These modifications were mostly reciprocally regulated (Ser^{422}, Ser^{418}) and located within or close to the PDZ domains of Dlg4-regions that have already been associated with fine-tuning Dlg4's molecular associations (Pedersen et al., 2017). Another example of a multiple phosphorylation site protein is Rims1, a scaffold element at the presynaptic active zone that regulates neurotransmitter release (Castillo et al., 2002). Rims1 contained six regulated phospho-sites during down-scaling and nine regulated sites during up-scaling. During the late phase of up-scaling, an increase in Ser^{592} phosphorylation of Rims1 (Ser^{413} in mice) was observed, which is associated with LTP and the recruitment of 14-3-3 adaptor proteins (Simsek-Duran et al., 2004).

In contrast to the highly regulated groups/phosphorylation hotspots, other phosphoproteins were exclusively regulated during one particular scaling phenotype. For example, phosphorylation events on the proteins complexin-1 (Cplx1) and Cplx2 of the presynaptic active zone showed a significant change only during the early phase of Bic-induced down-scaling. In addition, Bic-induced phosphorylation of two post-synaptic proteins— synaptic adhesion-like molecule 1/2 (Lrfn2 and Lrfn1), known to interact with central proteins of the postsynaptic density (e.g., Dlg4, Gria1 and Grin1; Ko et al., 2006)—was exclusively observed at 24 h. For TTX-induced up-scaling, three phosphorylation sites on Rims2, also a

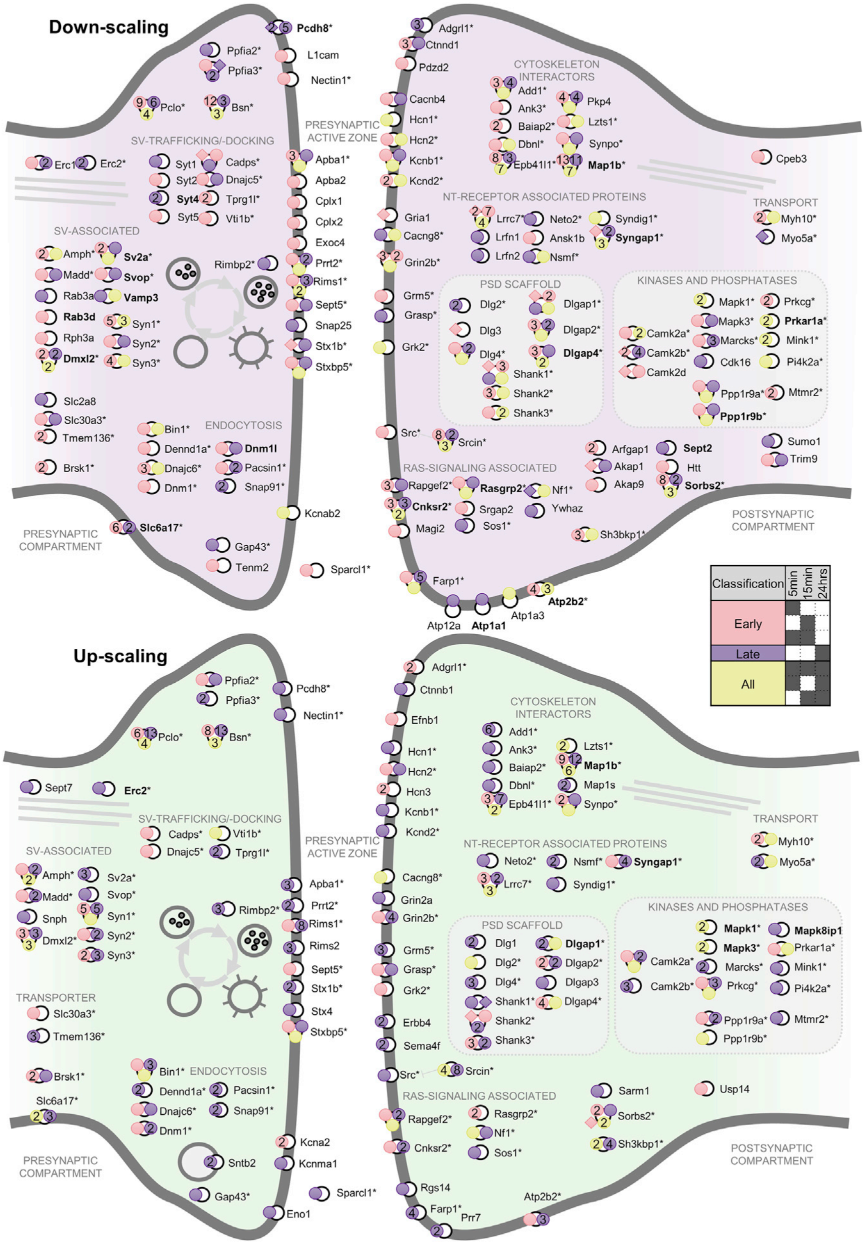

Figure 5. Activity-dependent protein phosphorylation in synaptic compartments

Differentially regulated phosphorylation sites on synaptic proteins during Bic-induced down-scaling (top) or TTX-induced up-scaling (bottom), displayed according to their synaptic localization. The regulated phosphorylation sites are displayed as circles on the respective protein and are colored according to their temporal categories (see legend). Proteins marked with an asterisk were phosphoregulated during both down- and up-scaling. Proteins highlighted in bold were regulated in protein abundance as well. In cases where multiple phosphopeptides covering the same phosphorylation site exhibited differences in their regulatory pattern, the classification of the singly phosphorylated peptide was displayed here.

protein of the presynaptic active zone, were regulated only at 24 h.

Lastly, we compared the phosphoregulation we observed during our synaptic scaling experiments to phosphoregulated proteins detected in previously published phosphoproteomics experiments. In general, the phosphoregulated proteins in our dataset were much more extensive but overlapped with the phosphoproteins detected following other forms of synaptic plasticity (Figures S7A and S7B), including LTP (Li et al., 2016), depolarization (Kohansal-Nodehi et al., 2016), or mGluR-dependent long-term depression (LTD) (van Gelder et al., 2020). Altogether, the phosphoproteins associated with synaptic scaling identified here captured between 60% and 75% of the phosphoproteins identified in these previous plasticity studies. In addition, we also identified ~40% of the phosphoproteins identified in a recent study examining the molecular time course of pathology associated with AD (Bai et al., 2020) (Figure S7B).

DISCUSSION

We generated a comprehensive, time-resolved map of the differential protein phosphorylation underlying homeostatic scaling in cultured cortical neurons. In response to the global activity manipulations that induce scaling, we detected both time-limited and persistent changes in over 3,300 phosphorylation events. We complemented our phosphoproteomic analyses with quantification of the total proteome and found a negligible contribution of protein abundance to the regulated phosphorylation events. Regulated phosphoproteins were significantly enriched for synaptic functions and cytoskeletal organization. As discussed below, a significant number of phosphoproteins were phosphorylated reciprocally during up- and down-scaling, reflecting the opposing scaling polarity.

Previous high-throughput studies investigated protein phosphorylation as a regulatory element in sleep (Brüning et al., 2019; Diering et al., 2017), LTP (Li et al., 2016), depolarization (Engholm-Keller et al., 2019; Kohansal-Nodehi et al., 2016), or mGluR-dependent LTD (van Gelder et al., 2020). A recent study explored AD stage-associated investigations (Bai et al., 2020). While the overlap of phosphoregulation with different studies on phosphorylation in synaptic plasticity was extensive, phosphoregulation in homeostatic scaling encompassed a broader set of targets, indicated by the great number of phosphoproteins exclusively regulated during scaling. Our dataset comprises a

broad and systematic study of protein phosphorylation during homeostatic synaptic scaling, examining phosphoproteomic changes that occur within minutes of activity offset and contrasting them to those that are responsible for the implementation of scaling detected 24 h later, as discussed below.

A key feature of a homeostatic response system is an output set point that is retargeted following an activity perturbation (Davis, 2006). To achieve homeostasis, an offset from the set point needs to be sensed and coupled to effector mechanisms to bring about synaptic scaling. In principle, the early activity-sensitive phosphorylation events we detected could comprise offset "sensors," and the late phosphorylation events could comprise "effectors."

We detected 282 and 1,039 regulated phosphorylation events unique to the early phase of up- or down-scaling, respectively; these modifications could be responsible for sensing the global change in activity. A subset of 110 events was shared between up- and down-scaling. These common sensor events could detect a sign-dependent or sign-independent activity set point offset. Indeed, we noted that 47% of these common early phosphoregulatory events were reciprocal in nature, manifest as either a decrease or an increase in phosphorylation, depending on the type of scaling. We noted that in early time points, down-scaling was associated with many more differential phosphorylation events than up-scaling. This could be due to rapid, Bic-evoked change in intracellular Ca^{2+} levels. Consistent with an early influx of Ca^{2+}, we detected the largest increase in phosphorylation of the Camk2a Thr^{286} site directly after 5 min Bic treatment. In addition, some Camk2a-associated phosphorylation events such as Ser^{16} of the cytoskeleton-interacting protein Stmn1 or the LTP-associated phosphorylation of Ser^{831} on the AMPA receptor subunit 1 (Gria1; exclusive phosphorylation; Lee, 2006) were uniquely detected during early down-scaling. During early TTX stimulation, the phosphorylation of Thr^{286} could not be reliably quantified, as the phosphorylated peptide species was not abundant. We also detected Camk2a-associated sites among the shared, bi-directional sensor sites, such as on the postsynaptic scaffold protein Shank3 (Ser^{1511}; Dosemeci and Jaffe, 2010). Also, the kinases ERK1/2, which can be secondarily activated by Ca^{2+}-dependent mechanisms (Thomas and Huganir, 2004; Zhu et al., 2002), exhibited an early (5 min) Bic-evoked increase in both phosphorylation sites of its activation loop (Thr^{203}/Tyr^{205}; Thr^{183}/Tyr^{185}).

Phosphorylation events that were present during the late phase of synaptic up- or down-scaling represent potential effectors—modifications that are required to express the homeostatic response. We detected 912 and 681 potential effector events unique to the late phase of up- and down-scaling, respectively. The number of common effector events shared between the opposite scaling groups was 149, with the majority (74%) exhibiting reciprocal, polarity-dependent regulation. Since synaptic scaling converges on changes of synaptic weight by changing the number of AMPA receptors in the postsynaptic membrane (O'Brien et al., 1998; Turrigiano et al., 1998), obvious effector proteins include glutamate receptors or receptor-associated molecules involved in receptor transport, surface retention, or functional modulation. While we identified regulation of Gria1 in the early phase of activity manipulation, we did not identify previously reported phosphorylation sites on the receptors during late phases of scaling, (e.g., Tyr^{876}, after 48 h up-scaling; Yong et al., 2020). Nevertheless, our data indicate a pronounced phosphomodulation of glutamate-receptor-associated and scaffolding proteins of the postsynaptic density. For example, we detected differential phosphorylation unique to the late phase of down-scaling on the proteins Lrfn1 and Lrfn2, known to induce clustering of excitatory proteins of the postsynaptic density (Ko et al., 2006) or involved in surface expression of AMPA receptor subunits (Seabold et al., 2008; Wang et al., 2006), respectively. Another example is the late up-scaling phosphoregulation of the protein Syndig1, which is known to interact with the AMPA receptor subunits Gria1 and Gria2 (Kalashnikova et al., 2010).

In contrast to time-sensitive phosphorylation events, we found that a considerable proportion (~25%) of initially regulated phospho-events were continuously switched either on or off (e.g., exhibited a persistent increase or decrease in phosphorylation) at the tested time points. They could function as set point offset sensors or scaling effectors, but whether there is a true persistence or a re-appearing/cyclic behavior in the regulation of these events cannot be determined. This set of phospho-events was associated with proteins of the postsynapse involved in organization of the cytoskeleton and, during down-scaling, in modulation of synaptic transmission as well. The observed persistent phosphorylation differs from the proteomic regulation throughout homeostatic scaling, where little overlap in the identity of the newly synthesized proteins of the early (2 h) and late phases (24 h) was observed (Schanzenbächer et al., 2018). Among the persistently regulated phospho-events, we found the Bic-evoked autophosphorylation sites of the kinases Camk2a (peak after 5 min Bic) and ERK1/2, suggesting both an early and late role for Ca^{2+}-sensitive kinases. For example, we detected the persistent phosphoregulation of Ser^{421} of the transcription factor Mecp2, which has been reported as a critical factor during synaptic scaling (Blackman et al., 2012). Ser^{421} is known to be phosphorylated by Camk2a in an activity-dependent manner, leading to induction of Bdnf transcription (Chen et al., 2003; Zhou et al., 2006). We found that Ser^{421} of Mecp2 was indeed phosphorylated during all time points following Bic treatment, and we detected a concomitant upregulation of Bdnf after 24 h Bic stimulation. Interestingly, a role for persistent kinase activity has been previously reported in several *in vivo* studies, suggesting a role in behavioral plasticity or long-term memory. For example, continuous hippocampal Camk2a autophosphorylation was observed up to 20 h after inhibitory avoidance training (Bambah-Mukku et al., 2014). In modeling studies, persistent autophosphorylation of Camk2a has been proposed to contribute to long-term information storage (Lisman and Goldring, 1988; Lisman and Raghavachari, 2015). Moreover, autophosphorylation of ERK1/2 kinases in long-term memory has been observed during spatial learning experiments with rodents (Blum et al., 1999; Selcher et al., 1999), and persistence in ERK1/2 activity was proposed in modeling studies of memory maintenance (Smolen et al., 2008).

Mechanistically speaking, the question arises of whether the phenotypically opposite poles of up- and down-scaling are mediated by reciprocal regulation of common proteins. Proteomic analyses have identified commonly regulated proteins in a

bi-directional manner during up- and down-scaling, but mostly divergence, suggesting the potential involvement of unique cellular pathways (Dörrbaum et al., 2020; Schanzenbächer et al., 2016). In this study, we identified 219 phosphorylation events that strictly follow a bi-directional pattern reflecting the opposing scaling polarities. We found that persistent phosphorylation events associated with one scaling paradigm were persistently and reciprocally regulated in the opposing scaling paradigm. Indeed, the majority (Bic, 68%; TTX, 85%) of the persistent phosphorylation events were associated with bi-directional regulation when directly compared to one another in a cluster analysis. The reciprocal and persistent phosphorylation events that we observed are predominantly associated with activity modulation of Camk2a or MAP kinases ERK1/2: autophosphorylation sites of these kinases and the differential kinase activity inferred from KSEA largely matched the same bi-directional, continuous pattern that was also verified by phospho-specific antibodies. The results of these analyses suggest persistence in catalytic kinase activity; however, a slower rate of dephosphorylation by phosphatases could also contribute. Characterizing the regulatory contribution of phosphatases remains a challenge (Fahs et al., 2016), as there are still only a few reports on substrate identifications (e.g., for the phospho-events of Ca^{2+}-sensitive phosphatase candidates matching the bi-directional profile we detected [Ppp3ca-c]). Reciprocal phosphorylation might also represent a polarity-specific tagging component allowing capture and/or retention of plasticity-related mRNAs or proteins driving downstream mechanisms, leading to scaling of corresponding polarity. Examples of phosphorylation sites that could serve as bi-directional switches are Ser^{422} or Ser^{516} on Dlg4 or Arhgef7, postsynaptic proteins involved in receptor-trafficking and signaling, respectively. In the late phase of scaling, phosphorylation of Ser^{418} of Dlg4 in the same reciprocal nature as Ser^{422} was also observed. With respect to protein structure, both sites are directly localized between the PDZ-3 and SH3 domain of Dlg4. These domains are known to enable the interaction with a broad spectrum of proteins (Kim and Sheng, 2004) and affect molecular association/protein localization (Pedersen et al., 2017). Overall, many synaptic proteins appeared as phosphorylation hotspots where phosphoregulation of different temporal categories converged, demonstrating that one protein can have multiple roles throughout the expression of the homeostatic response.

Taken together, our findings highlight protein phosphorylation as a major molecular driver following global activity manipulations for minutes to a day. Phosphomodulation during scaling induction was not only achieved by time-limited phosphorylation, but persistent and strictly bi-directional phosphorylation was also shown to play a prominent role in both phenotypes of homeostatic plasticity, connecting them mechanistically. In support of our data, we detected differential regulation of proteins as well as phosphorylation events that had already been reported in the context of synaptic plasticity, but we also identified new phosphoregulated candidates. The broad detection of distinct phosphorylation profiles provides insights into the fundamental processes that underlie activity sensing and scaling manifestation. These data thus provide resources for further candidate-based investigations into the role of long-lasting and/or bi-directional phosphorylation during homeostatic scaling and other forms of plasticity.

STAR★METHODS

Detailed methods are provided in the online version of this paper and include the following:

- KEY RESOURCES TABLE
- RESOURCE AVAILABILITY
  - Lead contact
  - Material availability
  - Data and code availability
- EXPERIMENTAL MODEL AND SUBJECT DETAILS
- METHOD DETAILS
  - MS-sample preparation and phosphopeptide enrichment
  - LC-MS/MS analysis
  - Western blot analysis
- QUANTIFICATION AND STATISTICAL ANALYSIS
  - MS-data processing
  - MS-data statistical analysis
  - Hierarchical clustering
  - Kinase substrate enrichment analysis
  - Phosphoprotein-interaction map
  - Comparison of proteome remodeling during synaptic scaling
  - Cell-type specific analyses of the regulated phosphoproteome
  - Synaptic phosphoproteome
  - Comparison to other phosphoproteomics studies

SUPPLEMENTAL INFORMATION

Supplemental information can be found online at https://doi.org/10.1016/j.celrep.2021.109583.

ACKNOWLEDGMENTS

We thank I. Bartnik, N. Fuerst, A. Staab, and C. Thum for the preparation of primary cell cultures; A. Dörrbaum for valuable advice on the statistical analyses; and F. Rupprecht for MS maintenance and assistance with data acquisition. We further thank A. Biever, C. Glock, and G. Tushev for the neuron-specific gene classifier. E.M.S. is funded by the Max Planck Society, an Advanced Investigator award from the European Research Council (grant 743216), DFG CRC 1080: Molecular and Cellular Mechanisms of Neural Homeostasis, and DFG CRC 902: Molecular Principles of RNA-based Regulation.

AUTHOR CONTRIBUTIONS

K.D. designed, conducted, and analyzed experiments and wrote the paper. J.D.L. and E.M.S. designed experiments, supervised the project, and co-wrote the paper.

DECLARATION OF INTERESTS

The authors declare no competing interests.

Received: February 12, 2021
Revised: June 15, 2021
Accepted: July 29, 2021
Published: August 24, 2021

REFERENCES

Aakalu, G., Smith, W.B., Nguyen, N., Jiang, C., and Schuman, E.M. (2001). Dynamic visualization of local protein synthesis in hippocampal neurons. Neuron *30*, 489–502.

Amir, R.E., Van den Veyver, I.B., Wan, M., Tran, C.Q., Francke, U., and Zoghbi, H.Y. (1999). Rett syndrome is caused by mutations in X-linked MECP2, encoding methyl-CpG-binding protein 2. Nat. Genet. *23*, 185–188.

Bai, B., Wang, X., Li, Y., Chen, P.-C., Yu, K., Dey, K.K., Yarbro, J.M., Han, X., Lutz, B.M., Rao, S., et al. (2020). Deep Multilayer Brain Proteomics Identifies Molecular Networks in Alzheimer's Disease Progression. Neuron *105*, 975–991.e7.

Bambah-Mukku, D., Travaglia, A., Chen, D.Y., Pollonini, G., and Alberini, C.M. (2014). A positive autoregulatory BDNF feedback loop via C/EBPβ mediates hippocampal memory consolidation. J. Neurosci. *34*, 12547–12559.

Bates, D., Mächler, M., Bolker, B.M., and Walker, S.C. (2015). Fitting linear mixed-effects models using lme4. J. Stat. Softw. *67* (*1*).

Bats, C., Groc, L., and Choquet, D. (2007). The interaction between Stargazin and PSD-95 regulates AMPA receptor surface trafficking. Neuron *53*, 719–734.

Benjamini, Y., and Hochberg, Y. (1995). Controlling the False Discovery Rate: A Practical and Powerful Approach to Multiple Testing. J. R. Stat. Soc. B *57*, 289–300.

Blackman, M.P., Djukic, B., Nelson, S.B., and Turrigiano, G.G. (2012). A critical and cell-autonomous role for MeCP2 in synaptic scaling up. J. Neurosci. *32*, 13529–13536.

Blum, S., Moore, A.N., Adams, F., and Dash, P.K. (1999). A mitogen-activated protein kinase cascade in the CA1/CA2 subfield of the dorsal hippocampus is essential for long-term spatial memory. J. Neurosci. *19*, 3535–3544.

Brüning, F., Noya, S.B., Bange, T., Koutsouli, S., Rudolph, J.D., Tyagarajan, S.K., Cox, J., Mann, M., Brown, S.A., and Robles, M.S. (2019). Sleep-wake cycles drive daily dynamics of synaptic phosphorylation. Science *366*, eaav3617.

Castillo, P.E., Schoch, S., Schmitz, F., Südhof, T.C., and Malenka, R.C. (2002). RIM1α is required for presynaptic long-term potentiation. Nature *415*, 327–330.

Chen, W.G., Chang, Q., Lin, Y., Meissner, A., West, A.E., Griffith, E.C., Jaenisch, R., and Greenberg, M.E. (2003). Derepression of BDNF transcription involves calcium-dependent phosphorylation of MeCP2. Science *302*, 885–889.

Cox, J., and Mann, M. (2008). MaxQuant enables high peptide identification rates, individualized p.p.b.-range mass accuracies and proteome-wide protein quantification. Nat. Biotechnol. *26*, 1367–1372.

Cox, J., Hein, M.Y., Luber, C.A., Paron, I., Nagaraj, N., and Mann, M. (2014). Accurate proteome-wide label-free quantification by delayed normalization and maximal peptide ratio extraction, termed MaxLFQ. Mol. Cell. Proteomics *13*, 2513–2526.

Czernik, A.J., Pang, D.T., and Greengard, P. (1987). Amino acid sequences surrounding the cAMP-dependent and calcium/calmodulin-dependent phosphorylation sites in rat and bovine synapsin I. Proc. Natl. Acad. Sci. USA *84*, 7518–7522.

Davis, G.W. (2006). Homeostatic control of neural activity: from phenomenology to molecular design. Annu. Rev. Neurosci. *29*, 307–323.

Diering, G.H., Nirujogi, R.S., Roth, R.H., Worley, P.F., Pandey, A., and Huganir, R.L. (2017). Homer1a drives homeostatic scaling-down of excitatory synapses during sleep. Science *355*, 511–515.

Dörrbaum, A.R., Alvarez-Castelao, B., Nassim-Assir, B., Langer, J.D., and Schuman, E.M. (2020). Proteome dynamics during homeostatic scaling in cultured neurons. eLife *9*, e52939.

Dosemeci, A., and Jaffe, H. (2010). Regulation of phosphorylation at the postsynaptic density during different activity states of Ca2+/calmodulin-dependent protein kinase II. Biochem. Biophys. Res. Commun. *391*, 78–84.

Ehlers, M.D. (2003). Activity level controls postsynaptic composition and signaling via the ubiquitin-proteasome system. Nat. Neurosci. *6*, 231–242.

Engholm-Keller, K., Waardenberg, A.J., Müller, J.A., Wark, J.R., Fernando, R.N., Arthur, J.W., Robinson, P.J., Dietrich, D., Schoch, S., and Graham, M.E. (2019). The temporal profile of activity-dependent presynaptic phospho-signalling reveals long-lasting patterns of poststimulus regulation. PLoS Biol. *17*, e3000170.

Fahs, S., Lujan, P., and Köhn, M. (2016). Approaches to Study Phosphatases. ACS Chem. Biol. *11*, 2944–2961.

Farley, J., and Schuman, E. (1991). Protein kinase C inhibitors prevent induction and continued expression of cell memory in Hermissenda type B photoreceptors. Proc. Natl. Acad. Sci. USA *88*, 2016–2020.

Ferrero, P., Said, M., Sánchez, G., Vittone, L., Valverde, C., Donoso, P., Mattiazzi, A., and Mundiña-Weilenmann, C. (2007). Ca2+/calmodulin kinase II increases ryanodine binding and Ca2+-induced sarcoplasmic reticulum Ca2+ release kinetics during β-adrenergic stimulation. J. Mol. Cell. Cardiol. *43*, 281–291.

Franceschini, A., Szklarczyk, D., Frankild, S., Kuhn, M., Simonovic, M., Roth, A., Lin, J., Minguez, P., Bork, P., von Mering, C., and Jensen, L.J. (2013). STRING v9.1: protein-protein interaction networks, with increased coverage and integration. Nucleic Acids Res. *41*, D808–D815.

Fukunaga, K., Stoppini, L., Miyamoto, E., and Muller, D. (1993). Long-term potentiation is associated with an increased activity of Ca2+/calmodulin-dependent protein kinase II. J. Biol. Chem. *268*, 7863–7867.

Glock, C., Biever, A., Tushev, G., Bartnik, I., Nassim-Assir, B., tom Dieck, S., and Schuman, E.M. (2020). The mRNA translation landscape in the synaptic neuropil. bioRxiv. https://doi.org/10.1101/2020.06.09.141960.

Greengard, P., Valtorta, F., Czernik, A., and Benfenati, F. (1993). Synaptic vesicle phosphoproteins and regulation of synaptic function. Science *259*, 780–785.

Hornbeck, P.V., Zhang, B., Murray, B., Kornhauser, J.M., Latham, V., and Skrzypek, E. (2015). PhosphoSitePlus, 2014: mutations, PTMs and recalibrations. Nucleic Acids Res. *43*, D512–D520.

Huke, S., and Bers, D.M. (2008). Ryanodine receptor phosphorylation at Serine 2030, 2808 and 2814 in rat cardiomyocytes. Biochem. Biophys. Res. Commun. *376*, 80–85.

Humphrey, S.J., James, D.E., and Mann, M. (2015). Protein Phosphorylation: A Major Switch Mechanism for Metabolic Regulation. Trends Endocrinol. Metab. *26*, 676–687.

Ibata, K., Sun, Q., and Turrigiano, G.G. (2008). Rapid synaptic scaling induced by changes in postsynaptic firing. Neuron *57*, 819–826.

Jang, S.S., Royston, S.E., Xu, J., Cavaretta, J.P., Vest, M.O., Lee, K.Y., Lee, S., Jeong, H.G., Lombroso, P.J., and Chung, H.J. (2015). Regulation of STEP61 and tyrosine-phosphorylation of NMDA and AMPA receptors during homeostatic synaptic plasticity. Mol. Brain *8*, 55.

Kalashnikova, E., Lorca, R.A., Kaur, I., Barisone, G.A., Li, B., Ishimaru, T., Trimmer, J.S., Mohapatra, D.P., and Díaz, E. (2010). SynDIG1: an activity-regulated, AMPA- receptor-interacting transmembrane protein that regulates excitatory synapse development. Neuron *65*, 80–93.

Kim, E., and Sheng, M. (2004). PDZ domain proteins of synapses. Nat. Rev. Neurosci. *5*, 771–781.

Ko, J., Kim, S., Chung, H.S., Kim, K., Han, K., Kim, H., Jun, H., Kaang, B.-K., and Kim, E. (2006). SALM synaptic cell adhesion-like molecules regulate the differentiation of excitatory synapses. Neuron *50*, 233–245.

Kohansal-Nodehi, M., Chua, J.J.E., Urlaub, H., Jahn, R., and Czernik, D. (2016). Analysis of protein phosphorylation in nerve terminal reveals extensive changes in active zone proteins upon exocytosis. eLife *5*, 1–25.

Kolde, R. (2019). pheatmap: Pretty Heatmaps. https://rdrr.io/cran/pheatmap/.

Lee, H.K. (2006). Synaptic plasticity and phosphorylation. Pharmacol. Ther. *112*, 810–832.

Li, J., Wilkinson, B., Clementel, V.A., Hou, J., O'Dell, T.J., and Coba, M.P. (2016). Long-term potentiation modulates synaptic phosphorylation networks and reshapes the structure of the postsynaptic interactome. Sci. Signal. *9*, rs8.

Lisman, J.E., and Goldring, M.A. (1988). Feasibility of long-term storage of graded information by the Ca2+/calmodulin-dependent protein kinase molecules of the postsynaptic density. Proc. Natl. Acad. Sci. USA *85*, 5320–5324.

Lisman, J., and Raghavachari, S. (2015). Biochemical principles underlying the stable maintenance of LTP by the CaMKII/NMDAR complex. Brain Res. *1621*, 51–61.

Malinow, R., Madison, D.V., and Tsien, R.W. (1988). Persistent protein kinase activity underlying long-term potentiation. Nature *335*, 820–824.

Marder, E., and Prinz, A.A. (2003). Current compensation in neuronal homeostasis. Neuron *37*, 2–4.

Martin, K.C., Michael, D., Rose, J.C., Barad, M., Casadio, A., Zhu, H., and Kandel, E.R. (1997). MAP kinase translocates into the nucleus of the presynaptic cell and is required for long-term facilitation in Aplysia. Neuron *18*, 899–912.

Miller, S.G., and Kennedy, M.B. (1986). Regulation of brain type II Ca2+/calmodulin-dependent protein kinase by autophosphorylation: a Ca2+-triggered molecular switch. Cell *44*, 861–870.

Nestler, E.J., and Greengard, P. (1983). Protein phosphorylation in the brain. Nature *305*, 583–588.

O'Brien, R.J., Kamboj, S., Ehlers, M.D., Rosen, K.R., Fischbach, G.D., and Huganir, R.L. (1998). Activity-dependent modulation of synaptic AMPA receptor accumulation. Neuron *21*, 1067–1078.

Pedersen, S.W., Albertsen, L., Moran, G.E., Levesque, B., Pedersen, S.B., Bartels, L., Wapenaar, H., Ye, F., Zhang, M., Bowen, M.E., and Strømgaard, K. (2017). Site-Specific Phosphorylation of PSD-95 PDZ Domains Reveals Fine-Tuned Regulation of Protein-Protein Interactions. ACS Chem. Biol. *12*, 2313–2323.

Sanderson, J.L., Scott, J.D., and Dell'Acqua, M.L. (2018). Control of Homeostatic Synaptic Plasticity by AKAP-Anchored Kinase and Phosphatase Regulation of Ca^{2+}-Permeable AMPA Receptors. J. Neurosci. *38*, 2863–2876.

Schanzenbächer, C.T., Sambandan, S., Langer, J.D., and Schuman, E.M. (2016). Nascent Proteome Remodeling following Homeostatic Scaling at Hippocampal Synapses. Neuron *92*, 358–371.

Schanzenbächer, C.T., Langer, J.D., and Schuman, E.M. (2018). Time- and polarity-dependent proteomic changes associated with homeostatic scaling at central synapses. eLife *7*, 1–20.

Seabold, G.K., Wang, P.Y., Chang, K., Wang, C.-Y., Wang, Y.-X., Petralia, R.S., and Wenthold, R.J. (2008). The SALM family of adhesion-like molecules forms heteromeric and homomeric complexes. J. Biol. Chem. *283*, 8395–8405.

Selcher, J.C., Atkins, C.M., Trzaskos, J.M., Paylor, R., and Sweatt, J.D. (1999). A necessity for MAP kinase activation in mammalian spatial learning. Learn. Mem. *6*, 478–490.

Shannon, P., Markiel, A., Ozier, O., Baliga, N.S., Wang, J.T., Ramage, D., Amin, N., Schwikowski, B., and Ideker, T. (2003). Cytoscape: a software environment for integrated models of biomolecular interaction networks. Genome Res. *13*, 2498–2504.

Sharma, K., Schmitt, S., Bergner, C.G., Tyanova, S., Kannaiyan, N., Manrique-Hoyos, N., Kongi, K., Cantuti, L., Hanisch, U.-K., Philips, M.-A., et al. (2015). Cell type- and brain region-resolved mouse brain proteome. Nat. Neurosci. *18*, 1819–1831.

Simsek-Duran, F., Linden, D.J., and Lonart, G. (2004). Adapter protein 14-3-3 is required for a presynaptic form of LTP in the cerebellum. Nat. Neurosci. *7*, 1296–1298.

Smolen, P., Baxter, D.A., and Byrne, J.H. (2008). Bistable MAP kinase activity: a plausible mechanism contributing to maintenance of late long-term potentiation. Am. J. Physiol. Cell Physiol. *294*, C503–C515.

Thiagarajan, T.C., Lindskog, M., and Tsien, R.W. (2005). Adaptation to synaptic inactivity in hippocampal neurons. Neuron *47*, 725–737.

Thomas, G.M., and Huganir, R.L. (2004). MAPK cascade signalling and synaptic plasticity. Nat. Rev. Neurosci. *5*, 173–183.

Turrigiano, G.G. (2008). The self-tuning neuron: synaptic scaling of excitatory synapses. Cell *135*, 422–435.

Turrigiano, G. (2012). Homeostatic synaptic plasticity: local and global mechanisms for stabilizing neuronal function. Cold Spring Harb. Perspect. Biol. *4*, a005736.

Turrigiano, G.G., Leslie, K.R., Desai, N.S., Rutherford, L.C., and Nelson, S.B. (1998). Activity-dependent scaling of quantal amplitude in neocortical neurons. Nature *391*, 892–896.

Tyanova, S., Temu, T., Sinitcyn, P., Carlson, A., Hein, M.Y., Geiger, T., Mann, M., and Cox, J. (2016). The Perseus computational platform for comprehensive analysis of (prote)omics data. Nat. Methods *13*, 731–740.

Ubersax, J.A., and Ferrell, J.E., Jr. (2007). Mechanisms of specificity in protein phosphorylation. Nat. Rev. Mol. Cell Biol. *8*, 530–541.

Vallejo, D., Codocedo, J.F., and Inestrosa, N.C. (2017). Posttranslational Modifications Regulate the Postsynaptic Localization of PSD-95. Mol. Neurobiol. *54*, 1759–1776.

van Gelder, C.A., Penning, R., Veth, T., Catsburg, L.A., Hoogenraad, C.C., MacGillavry, H.D., and Altelaar, M. (2020). Temporal quantitative proteomics of mGluR-induced protein translation and phosphorylation in neurons. Mol. Cell. Proteomics *19*, 1952–1968.

Vizcaíno, J.A., Côté, R.G., Csordas, A., Dianes, J.A., Fabregat, A., Foster, J.M., Griss, J., Alpi, E., Birim, M., Contell, J., et al. (2013). The PRoteomics IDEntifications (PRIDE) database and associated tools: status in 2013. Nucleic Acids Res. *41*, D1063–D1069.

Wang, C.-Y., Chang, K., Petralia, R.S., Wang, Y.X., Seabold, G.K., and Wenthold, R.J. (2006). A novel family of adhesion-like molecules that interacts with the NMDA receptor. J. Neurosci. *26*, 2174–2183.

Wiredja, D.D., Koyutürk, M., and Chance, M.R. (2017). The KSEA App: a web-based tool for kinase activity inference from quantitative phosphoproteomics. Bioinformatics *33*, 3489–3491.

Yong, A.J.H., Tan, H.L., Zhu, Q., Bygrave, A.M., Johnson, R.C., and Huganir, R.L. (2020). Tyrosine phosphorylation of the AMPA receptor subunit GluA2 gates homeostatic synaptic plasticity. Proc. Natl. Acad. Sci. USA *117*, 4948–4958.

Zhou, Z., Hong, E.J., Cohen, S., Zhao, W.N., Ho, H.Y., Schmidt, L., Chen, W.G., Lin, Y., Savner, E., Griffith, E.C., et al. (2006). Brain-specific phosphorylation of MeCP2 regulates activity-dependent Bdnf transcription, dendritic growth, and spine maturation. Neuron *52*, 255–269.

Zhu, J.J., Qin, Y., Zhao, M., Van Aelst, L., and Malinow, R. (2002). Ras and Rap control AMPA receptor trafficking during synaptic plasticity. Cell *110*, 443–455.

STAR★METHODS

KEY RESOURCES TABLE

REAGENT or RESOURCE	SOURCE	IDENTIFIER
Antibodies		
Camk2a	Invitrogen	Cat# 13-7300; RRID: AB_2533032
phospho-Camk2a (pT^{286})	Cell Signaling Technology	Cat# 12716; RRID: AB_2713889
Mapk1/Mapk3	Cell Signaling Technology	Cat# 4696; RRID: AB_390780
phospho-Mapk1/Mapk3 (pT^{183}-pY^{185}/ pT^{203}-pY^{205})	Cell Signaling Technology	Cat# 4370; RRID: AB_2315112
Goat anti-mouse IRDye 680	LI-COR	Cat# 926-68020; RRID: AB_10706161
Goat anti-rabbit IRDye 800	LI-COR	Cat# 926-32211; RRID: AB_621843
Critical commercial assays		
BCA protein assay kit	ThermoFisher Scientific	Cat# 23225
TiO_2 Phosphopeptide Enrichment Kit	ThermoFisher Scientific	Cat# A32993
Chemicals, peptides and recombinant proteins		
Neurobasal-A	Life Technologies	Cat# 10888022
B27	GIBCO	Cat# 17504044
GlutaMax	GIBCO	Cat# 35050038
Bicuculline methochloride	Tocris Bioscience	Cat# 0131
Tetrodotoxin citrate	Tocris Bioscience	Cat# 1069
PhosStop phosphatase inhibitor	Roche, Sigma-Aldrich	Cat# 4906845001
cOmplete protease inhibitor cocktail (EDTA-free)	Roche, Sigma-Aldrich	Cat# 4693159001
Benzonase	Sigma-Aldrich	Cat# E1014
Iodoacetamide	ThermoFisher Scientific	Cat# A39271
Sequencing grade modified trypsin	Promega	Cat# V5111
NuPAGE MES SDS running buffer (20x)	Life Technologies	Cat# NP0002
Intercept blocking buffer (TBS)	LI-COR	Cat# 927-60001
Deposited data		
Synaptic scaling phosphoproteomics raw data	this study	PRIDE: PXD021834
Software and algorithms		
MaxQuant (1.6.6.0)	(Cox and Mann, 2008)	RRID:SCR_014485
Perseus (1.6.2.3)	(Tyanova et al., 2016)	RRID:SCR_015753
pheatmap R package	(Kolde, 2019)	RRID:SCR_016418
KSEAapp R package	(Wiredja et al., 2017)	CRAN.R-project.org/package=KSEAapp/
lme4 R package	(Bates et al., 2015)	RRID:SCR_015654
Cytoscape (3.7.2)	(Shannon et al., 2003)	RRID:SCR_003032
STRING database	(Franceschini et al., 2013)	RRID:SCR_005223
PhosphoSitePlus database	(Hornbeck et al., 2015)	RRID:SCR_001837
Other		
S-Trap (mini columns)	ProtiFi	https://protifi.com/pages/s-trap
Sep-Pak cartridge C18 (50mg sorbent)	Waters	Cat# 186000308
NuPAGE 4-12% Bis-Tris Gel	ThermoFisher Scientific	Cat# NP0322BOX
Immobilon-FL transfer membrane (PVDF)	Merck Milipore	Cat# IPFL00010

RESOURCE AVAILABILITY

Lead contact

Further information and requests for resources and reagents should be directed to and will be fulfilled by the lead contact, Erin M. Schuman (erin.schuman@brain.mpg.de).

Material availability

This study did not generate new unique reagents.

Data and code availability

Mass spectrometry data associated with this manuscript have been uploaded to the PRIDE repository and are available with the dataset identifier PRIDE: PXD021834 (RRID:SCR_003411; Vizcaíno et al., 2013). The scripts used in this paper as well as any additional information required to reanalyze the data reported in this paper are available upon request.

EXPERIMENTAL MODEL AND SUBJECT DETAILS

Dissociated cortical neurons were prepared and maintained as previously described for hippocampus neurons (Aakalu et al., 2001). Cortices from postnatal day one old rat pups of either sex (RRID:RGD_734476; strain Sprague-Dawley) were dissected, dissociated by incubating with L-cysteine-papain solution at 37°C and plated onto 10 cm Petri dishes (MatTek, Ashland, MA) previously coated with poly-D-lysine. Cultured cells were kept in Neurobasal-A medium (Invitrogen, Carlsbad, CA) supplemented with B-27 (Invitrogen) and Glutamax (Invitrogen) at 37°C and 5% CO_2 for 19-20 days.

All experiments complied with national animal care guidelines and the guidelines issued by the Max Planck Society and were approved by local authorities.

METHOD DETAILS

MS-sample preparation and phosphopeptide enrichment

Nine dishes (3 million cells/ dish) were prepared for each experiment. Three dishes each were treated with either 20 μM Bicuculline, 1 μM Tetrodotoxin or no drug (control) for 5 min, 15 min or 24 hr. Afterward, the cells were harvested by briefly washing with ice-cold DPBS (Invitrogen) supplemented with protease inhibitor cocktail (cOmplete EDTA-free; Roche, Basel, Switzerland) and phosphatase inhibitors (PhosStop; Roche), followed by scraping and pelleting by centrifugation.

Cell pellets were lysed using lysis buffer (5% SDS, 25 mM Tris, pH 7.55, supplemented with protease and phosphatase inhibitor), and then disrupted with a pipette and four sonication cycles for 30 s. Lysates were incubated with Benzonase (1 μl; 250 units/mL stock solution; Sigma, St. Louis, MO) for 10 min at room temperature. To clear debris from the samples, they were centrifuged for 8 min at 13,000 x g. Protein concentration was determined by a BCA assay (ThermoFisher Scientific, Waltham, MA). The samples were diluted 1:5 prior to the assay to minimize interference of the high detergent concentration.

For bottom-up MS analysis, protein digestion was performed according to an adapted version of the suspension trapping protocol as described by the manufacturer (S-Trap, ProtiFi, Huntington, NY). In brief, 350 μg of protein in lysis buffer was reduced by DTT addition in a final concentration of 20 mM for 10 min. Then proteins were alkylated using iodoacetamide at a final concentration of 40 mM and incubated for 30 min at room temperature in the dark. Afterward, the sample was acidified by addition of phosphoric acid to a final concentration of 1.2%. Binding buffer (90% methanol, 50 mM TRIS, pH 7.55) was added in a 1:7 lysate to buffer ratio. The mixture was loaded onto the S-Trap filter (mini) by centrifugation for 30 s at 4,000 x g in 450 μl-steps and washed with 400 μl binding buffer for four times. Sequencing-grade trypsin (Promega, Madison, WI) was added in 150 μl digestion buffer (40 mM ammonium bicarbonate) in an enzyme-to-protein ratio of 1:50. The protease buffer was briefly (1-3 s) spun into the trap; solution passing the filter was re-added on top. Digestion was carried out overnight at room temperature under gentle agitation and in a humidified chamber to prevent filters from drying out. To elute peptides, the filter was rinsed in three consecutive steps by centrifugation at 1,000 x g for 60 s starting with 80 μl digestion buffer and two 80 μl washes with 0.2% formic acid (FA) in MS grade water.

After digestion, the peptides were desalted using C_{18}-SepPak columns (50 mg sorbent; Waters, Milford, MA) as previously described (Schanzenbächer et al., 2016). Desalted peptides were separated for analysis of the total proteome (5% v/v) and subsequent enrichment for phosphorylated peptides (95% v/v). All samples were dried *in vacuo* using a Speed Vac (Eppendorf, Hamburg, Germany) at room temperature and stored at −20°C until LC- MS analysis or further use. Enrichment for phosphorylated peptides was performed using Titanium dioxide beads (TiO_2; kit: #432993; ThermoFisher Scientific) as described in the manufacturer's protocol. Eluted peptides were dried *in vacuo* at room temperature and stored at −20°C until LC-MS analysis.

Each experiment was carried out in four independent biological replicates.

LC-MS/MS analysis

Dried peptides or phosphorylated peptides were reconstituted in 5% acetonitrile (ACN) with 0.1% FA or 2% ACN with 0.1% FA, respectively. Peptides were loaded onto a C_{18}-PepMap 100 trapping column (particle size 3 μm, L = 20 mm) and separated on a

C_{18}-EasySpray analytical column (particle size = 2 μm, ID = 75 μm, L = 50 cm, ThermoFisher Scientific) using a nano-HPLC (Dionex U3000 RSLCnano). Temperature of the column oven was maintained at 55°C.

Trapping was carried out for 6 min with a flow rate of 6 μl/min using loading buffer (100% H_2O with 0.05% triflouroacetic acid). Peptides were separated by a gradient of water (buffer A: 100% H_2O and 0.1% FA) and acetonitrile (buffer B: 80% ACN, 20% H_2O and 0.1% FA) with a constant flow rate of 300 nL/min. The gradient for unmodified peptides went from 4% to 48% buffer B in 180 min, the gradient for phosphorylated peptides from 4% to 30% buffer B in 110 min and to 45% buffer B in 10 min. All solvents were LC-MS grade and purchased from Riedel-de Häen/Honeywell (Seelze, Germany).

Eluting peptides were analyzed in a data-dependent acquisition mode on a Fusion Lumos mass spectrometer (ThermoFisher Scientific) coupled to the nano-HPLC (Dionex U3000 RSLCnano) by an EASY Spray ESI source. MS1 survey scans were acquired over a scan-range of 350 to 1400 mass-to-charge ratio (m/z) in the Orbitrap detector (resolution (R) = 120k, automatic gain control (AGC) = 2e5 and maximum injection time: 50 ms,). Sequence information was acquired by a "top speed" MS2 method with a fixed cycle time of 2 s for the survey and after MS/MS scans. MS2 scans were generated from the most abundant precursors with a minimum intensity of 5e3 and charge states from two to five. Selected precursors were isolated in the quadrupole using a 1.4 Da window and fragmented using higher-energy C-trap dissociation (HCD) at 30% normalized collision energy. For MS2, an AGC of 1e4 and a maximum injection time of 300 ms were used. Resulting fragments were detected in the ion trap using the rapid scan rate (AGC = 1e4, maximum injection time = 300 ms). Dynamic exclusion was set to 30 s with a mass tolerance of 10 parts per million (ppm). All LC- and MS-parameters are listed in the Table S1. Each sample was measured in triplicate LC-MS/MS runs.

Western blot analysis

Primary cortical cultures were prepared and maintained as described above. After 19 DIV, cells were incubated with either 20 μM Bicuculline, 1 μM Tetrodotoxin or no drug (control) for 5 minutes or 24 hr. Cell lysates were prepared as described and equal protein amounts were loaded onto 4% to 12% Bis-Tris NuPAGE gels (ThermoFisher Scientific). After electrophoreses, proteins were transferred to a PVDF membrane (Immobilon-FL; Merck Millipore, Billerica, MA). Immunoblotting was performed with primary antibodies against Camk2a phosphorylated at pT^{286} (1:1000, Cell Signaling Technology Ref.: 12716), total Camk2a (1:1000, Invitrogen Ref.: 13-7300), Mapk1/ Mapk3 phosphorylated at pT^{183}-pY^{185}/ pT^{203}-pY^{205} (1:1000, Cell Signaling Technology, Ref.: 4370) or total Mapk1/ Mapk3 (1:1000, Cell Signaling Technology, Ref.: 4696). Secondary antibodies, anti-mouse (1:15,000, Ref.: 926-68020) and anti-rabbit (1:15,000, Ref.: 926-3211) were purchased from LI-COR (Lincoln, NE). Densiometric quantification was conducted using LI-COR Image Studio Lite (RRID:SCR_013715). All experiments were performed in three independent biological replicates.

QUANTIFICATION AND STATISTICAL ANALYSIS

MS-data processing

MS raw data were processed using the MaxQuant software (ver. 1.6.6.0; RRID:SCR_014485; Cox and Mann, 2008) with customized parameters for the Andromeda search engine. For all searches, spectra were matched to the *Rattus norvegicus* database downloaded from the UniProtKB (RRID:SCR_004426; Proteome_ID: UP000002494; downloaded on 23 August 2019), a contaminant and decoy database. Tryptic peptides with a minimum length of seven amino acids and a maximum of two missed cleavage sites were included. Precursor mass tolerance was set to 4.5 ppm and fragment ion tolerance to 0.5 Da. Carboxyamidomethylation of cysteine residues was set as a static modification. Acetylation (Protein-N-term.) and oxidation of methionine residues were assigned as variable modifications. Analysis of the phosphoproteome included the assignment of phosphorylation of serine, threonine and tyrosine residues as variable modification. With the use of a decoy strategy, a false discovery rate (FDR) below 1% at protein, peptide and modification level was applied. The "match between runs" option was enabled (matching time window = 0.7 min, alignment time window = 20 min). Only proteins identified by at least one unique peptide were considered for further analysis. Label-free quantification of proteins was performed by pairwise ratio determination using at least two common peptides in at least three consecutive full scans (Cox et al., 2014). For all details on the parameters for raw-data processing in MaxQuant see the Table S2.

MS-data statistical analysis

Four independent biological replicates measured in triplicates were processed using the Perseus software (ver. 1.6.2.3; RRID:SCR_015753; Tyanova et al., 2016). MaxQuant protein results (total proteome: proteinGroups.txt) and results of the phosphorylation site table (phosphoproteome: phospho(STY)sites.txt) were filtered for contaminants and reverse (decoy) database hits. The phosphoproteomic data were further filtered for localization probability of the phosphorylated residue greater than 75% to trace the modification to one particular residue. Then the phosphorylation site table was rearranged according to multiplicity, i.e., an expansion of entries so that the number of phosphorylation sites per peptide was formatted as separate rows. Species of this modified table were called phosphorylation events, since there can be more than one entry for a particular phosphorylation site, e.g., derived from a singly, doubly or triply phosphorylated peptide species. Protein LFQ intensities and phosphorylation event intensities were log_2-transformed and normalized according to the sample's median intensity.

For quantitative comparisons at each time point (5 min, 15 min and 24 hr), only treatment-control pairs of proteins or phosphorylation events, which could be quantified in all four biological replicates in at least one technical replicate, were considered for further statistical analysis. Additional valid value filtering required 50% valid entries across each pairwise data matrix. We used the lme4

package in the statistical computing software R to perform a linear mixed effect analysis (RRID:SCR_015654; Bates et al., 2015). Differential regulation of treatment versus control was investigated using a model where the treatment was considered the fixed effect in question and the biological replicate was entered as a possible random effect, similar to previously reported strategies (Dörrbaum et al., 2020). P values were calculated by likelihood ratio tests of the model including the effect of interest against the model without it. To correct for multiple testing, Benjamini-Hochberg correction was applied with an FDR cut-off < 0.01 (Benjamini and Hochberg, 1995).

Hierarchical clustering

For hierarchical clustering analysis, log_2-tranformed fold changes of treatment against control intensities of significantly regulated phosphorylation events were used. The particular segmentation of the data in the clusters was stated in the corresponding figure legends. Distance measure to cluster the rows was Euclidean distance. Clustering was performed using the "ward.D2" agglomeration algorithm. Visualization using this metrics was done using the pheatmap package in the statistical computing software R (RRID:SCR_016418; Kolde, 2019).

Kinase substrate enrichment analysis

To match differentially regulated protein phosphorylation sites to their reported kinases, kinase substrate enrichment analysis was carried out using the KSEAapp package in the statistical computing software R (Wiredja et al., 2017). Site-specific information on the kinase-substrate pairs was downloaded from PhosphositePlus.org database (on 5th November 2019; RRID:SCR_001837; Hornbeck et al., 2015) and filtered for species-specific entries to *Rattus norvegicus*. NetworKIN predictions were excluded. For survey, a minimum number of one substrate of a kinase was set. Kinase z-scores representing the normalized score of each kinase weighted by the number of identified substrates were calculated. Multiple hypothesis testing to assess a p value for the kinase z-score was corrected using the Benjamini-Hochberg method (Benjamini and Hochberg, 1995). The log_2-transformed fold change of this site is reported and if a particular substrate phosphorylation site was detected across multiple phosphorylated peptide, its average is used in the algorithm.

Phosphoprotein-interaction map

Proteins harboring a phosphorylation event that was regulated in four out of six conditions (Figure 3) were analyzed for protein interactions using their gene identifiers and the STRING database (RRID:SCR_005223; Franceschini et al., 2013). Interactions with a confidence > 0.4 (combined score) were included in the analysis. Interactions solely based on textmining as a source were excluded. Networks were exported and visualization was performed using the software Cytoscape (ver. 3.7.2; RRID:SCR_003032; Shannon et al., 2003).

Comparison of proteome remodeling during synaptic scaling

The differentially regulated proteome characterized in this study was compared to results of another system-wide proteomics investigation of homeostatic scaling. For this purpose, we analyzed the data published by Dörrbaum et al. (2020) where primary-cultured hippocampal neurons were subjected to Bic or TTX treatment for one, three and seven days or left untreated. For direct comparison, only data of the control condition and the 24 hr treatments were considered. Quantification of proteins needed to have at least one valid treatment-control pair in all three biological replicates. For statistical evaluation, a linear mixed effects model was used as described in Dörrbaum et al., 2020. In brief, treatment was set as fixed effect in question, biological replicate and peptide identity nested into biological replicate were set as random effects. This resulted in 150 and 239 proteins that significantly changed in abundance in response to Bic or TTX treatment (Benjamini-Hochberg correction, FDR < 0.01, Benjamini and Hochberg, 1995). Proteins quantified in both studies exhibiting significant regulation at least one or both of them were used for correlation analysis.

Cell-type specific analyses of the regulated phosphoproteome

To assess contribution of different cell-types known to occur in mixed cultures of primary brain areas, we first compared the regulated phosphoproteome to established marker proteins for neurons, microglia, oligodendrocytes and astrocytes (Sharma et al., 2015). For this purpose, the top 50 differentially expressed proteins as identified by Sharma et al. were selected based on their abundance fold change (log_2-scaled cell-type specific expression over all cells) and matched to the regulated phosphoproteome using their gene name. Furthermore, a systematic approach to identify excitatory neuron-enriched proteins was performed by matching a previously reported neuron-enriched classifier (Glock et al., 2020) to the regulated phosphoproteome (by gene names). The classifier was generated to bioinformatically filter for transcripts preferentially expressed in excitatory neurons: Glock et al. curated the markers by focusing on neuronal genes and subtracting the contribution of transcripts from other cell types identified in different types of experiments: RNA sequencing of neuron-enriched versus glia-enriched cultures, Rpl22-HA-IP from hippocampi of Camk2a-Cre- or microdissected somata and neuropil of Wfs1-Cre-Ribotag mice.

Synaptic phosphoproteome

Proteins of the synaptic phosphoproteome (Figure 5) were selected if they matched a synaptic GO term for the cellular compartment (GOCC) and carried at least one significantly regulated phosphorylation site. Localization within the synapse was derived from the

UniProtKB database information on subcellular localization. If there was more than one phosphorylation event for a particular phosphorylation site on a protein, the site-specific, temporal categorization (early, late or all) was derived from the species with the lowest multiplicity to achieve highest residue resolution. Phosphorylation events which were termed and highlighted as “exclusive” for a certain time point were excluded from statistical analysis as they were quantified reliably only in one treatment group but not in the control or other treatment groups. Nonetheless, the peptides were visualized in the overview (depicted as a square) as this subset were reliably quantified in all four biological replicates of the condition in question and not detected in all biological replicates of any other possible conditions.

Comparison to other phosphoproteomics studies

To compare phosphoregulation associated with synaptic plasticity (van Gelder et al., 2020; Kohansal-Nodehi et al., 2016; Li et al., 2016), we extracted the significantly phosphoregulated species of the studies reported in different mass-spec based, high-throughput analyses. Significance was determined by the statistics and parameters reported in each study. We finally compared phosphomodulation on the level of phosphoregulated proteins by matching the studies via a species-neutral ID, gene names (Figures S7A and S7B).

Cell Reports, Volume *36*

Supplemental information

Dynamic bi-directional phosphorylation events associated with the reciprocal regulation of synapses during homeostatic up- and down-scaling

Kristina Desch, Julian D. Langer, and Erin M. Schuman

Supplemental Figures

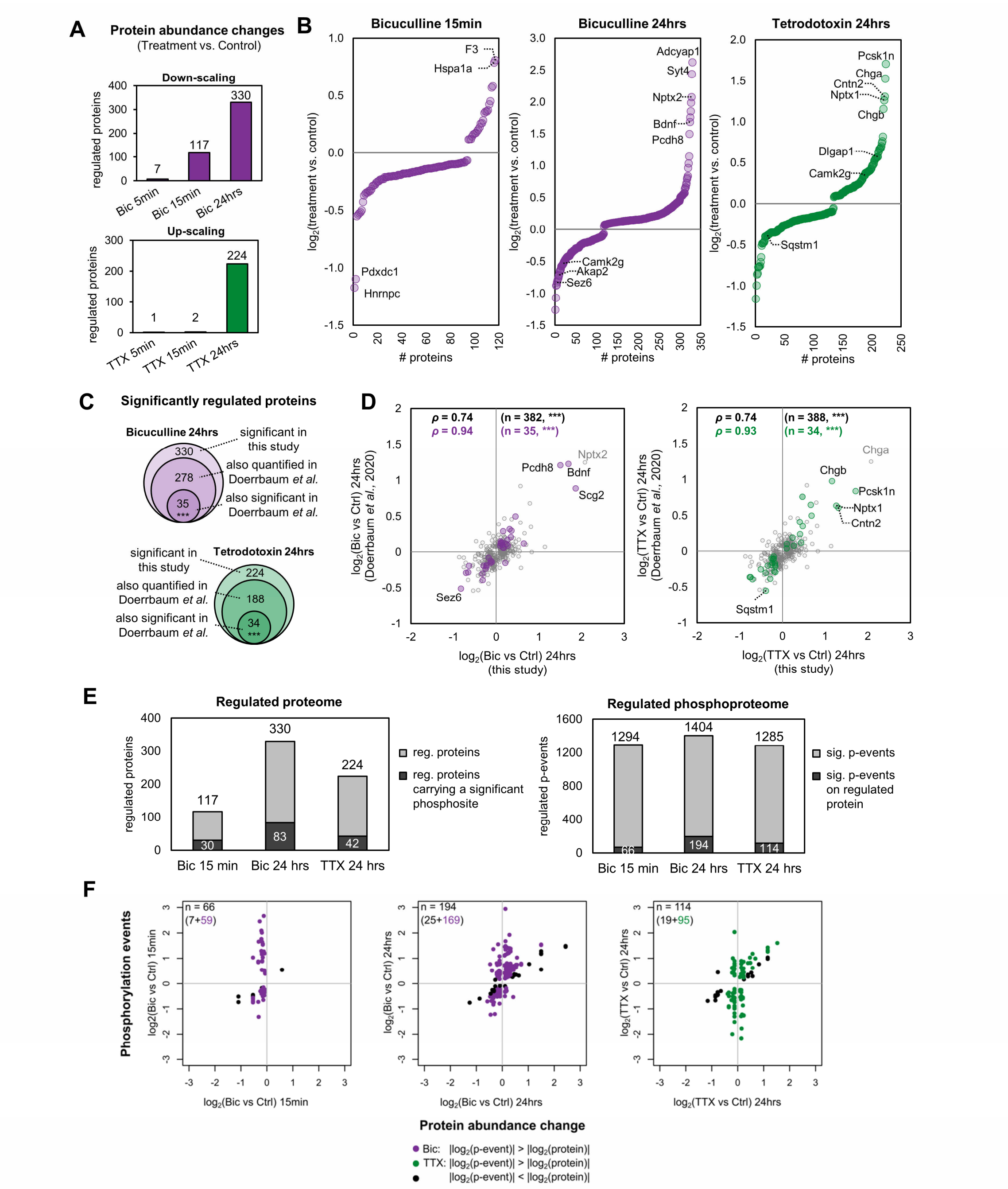

Figure S1 – Remodeling of the cortical proteome during homeostatic synaptic scaling. Related to Figure 1.

(A) Bar chart depicts the number of significantly regulated proteins at each time point of Bic or TTX treatment compared to the control group (Benjamini-Hochberg correction; FDR < 0.01).

(B) Rank-ordered $\log_2$ fold changes of the regulated proteins (treatment vs. control) for the three time points showing the greatest changes in protein abundance: Bic treatment for 15 min and 24 hrs and TTX treatment for 24 hrs.

(C) Comparison of the significantly regulated proteins from this experiment to Doerrbaum *et al.* (see Methods), 278 of the 330 proteins regulated by Bic and 188 of 224 regulated by TTX were also identified in their dataset. Significantly more proteins were regulated in experiments of both studies after 24 hours of Bic and TTX treatment than by chance (Fisher‘s exact test; *** p <0.001; fold enrichment (FE): $FE_{Bic} = 3.4$, $FE_{TTX} = 2.9$).

(D) Pearson correlations of the $\log_2$ fold changes (treatment vs. control) of the proteins quantified in both studies were significant (*** $p < 0.001$): The Pearson correlation coefficient (ρ) of the overlapping proteins significantly regulated after 24 hours Bic or TTX treatment in at least one of the studies was 0.74 in both cases (n_{Bic} = 382, n_{TTX} = 388; grey). The Pearson correlation coefficient of the overlapping proteins exhibiting significant regulation in both studies was 0.94 or 0.93, respectively (n_{Bic} = 35, n_{TTX} = 34; purple/ green).

(E) Bar plot (left) shows the significantly regulated proteins after Bic treatment for 15 min and 24 hrs or TTX treatment for 24 hrs, highlighting the proportion of regulated proteins that carry at least one significantly regulated phosphorylation site in the same condition in a dark shade. Bar plot (right) displays the significantly regulated phosphorylation events after Bic treatment for 15 min and 24 hrs and TTX treatment for 24 hrs, highlighting the proportion of events that are located on a protein exhibiting significant abundance changes in the same condition.

(F) Scatterplot shows the $\log_2$ fold changes of the overlapping subset of regulated phosphorylation events versus the $\log_2$ fold changes of the corresponding regulated protein. In cases where the change of the phosphorylation event was greater than that of the protein it is located on, the dots are highlighted by color. Black dots indicate a greater change in protein abundance. The majority of phosphorylation events were found to be much more dynamic in all three conditions. The amplitude of regulation on phosphorylation level exceeded those on proteome level. This indicates the activity-dependent changes in phosphopeptide abundance observed in our dataset mostly arise from differential phosphorylation rather than proteome remodeling.

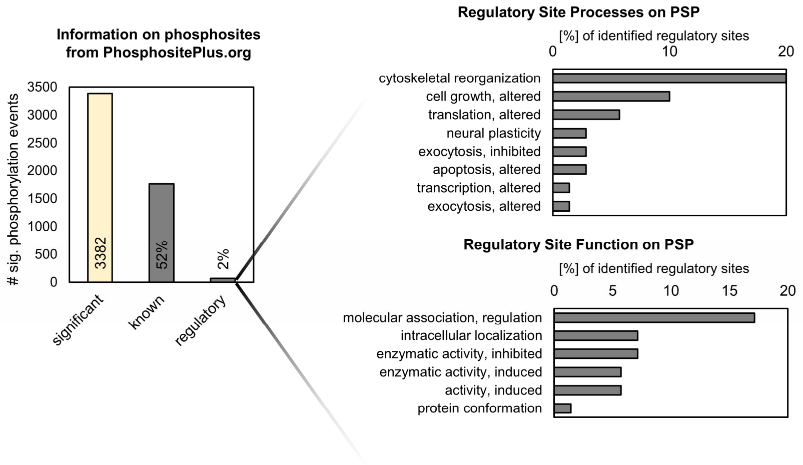

Figure S2 – Annotation of regulated phosphorylation events across all experimental groups. Related to Figure 1.

Significant change in phosphorylation was detected for 3,382 different phosphorylation events regulated along all experimental conditions (Benjamini-Hochberg correction; FDR <0.01). Information on the sites was assigned using the annotations of the PhosphoSitePlus database (Hornbeck et al., 2015). About 50% of the activity-dependent events had been identified in previous experiments (known) and 2% had a regulatory annotation (regulatory). On the left, the terms assigned to characterize the process (top) or function (bottom) of the regulatory sites are listed.

A

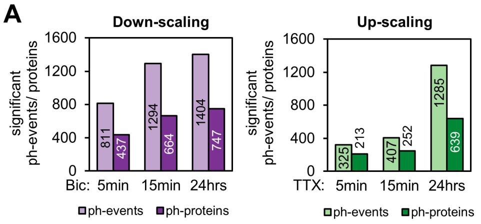

B

Condition		Av. reg. sites/ prot.
Bic	5min	1.77
	15min	1.84
	24hrs	1.80
TTX	5min	1.46
	15min	1.52
	24hrs	1.88

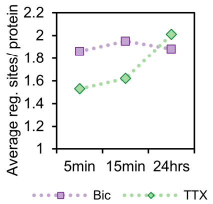

C

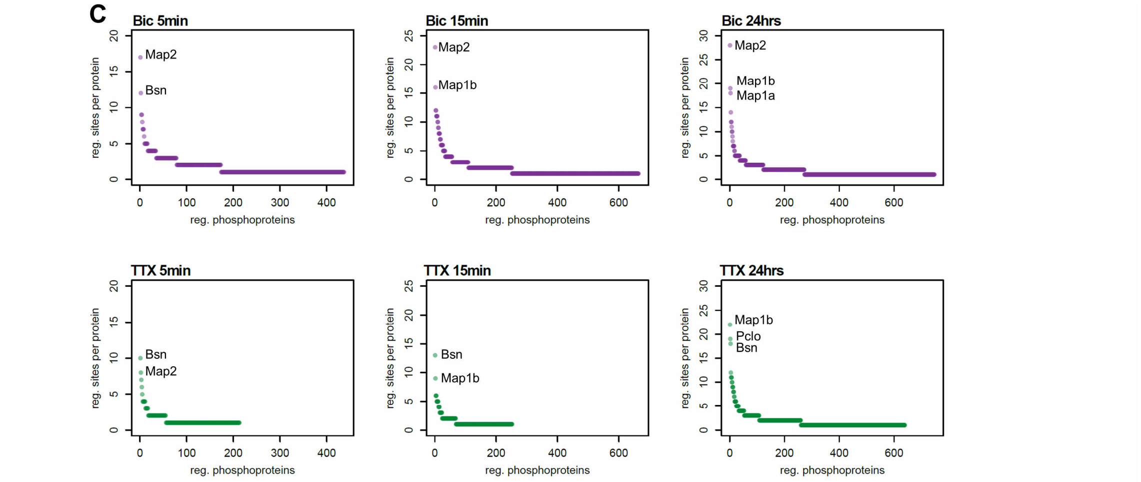

Figure S3 – Activity-sensitive multi-site phosphorylation. Related to Figure 1 and 2.

(A) Temporal distribution of regulated phosphorylation events and unique phosphoproteins. The bar charts represent the number of significantly regulated phospho-events and unique phosphoproteins harboring at least one of these events for each time point of down- (left) or up-scaling (right). Phosphorylation events exhibiting significant regulation comparing treatment versus control condition (Benjamini-Hochberg correction; FDR < 0.01) are shown in a lighter shade, unique phosphoproteins are depicted in a darker shade.

(B) Average phospho-regulation per protein. Table and diagram indicate the average number of phosphorylation sites per protein regulated during 5 min, 15 min or 24 hrs of homeostatic up- or down-scaling (Benjamini-Hochberg correction; FDR < 0.01).

(C) Distribution of regulated phosphorylation sites per protein. Plots show the number of regulated phosphorylation sites per protein during down-scaling (top row) or up-scaling (bottom row) separated by time point. Proteins with the highest number of sites in each condition were labeled according to their gene name.

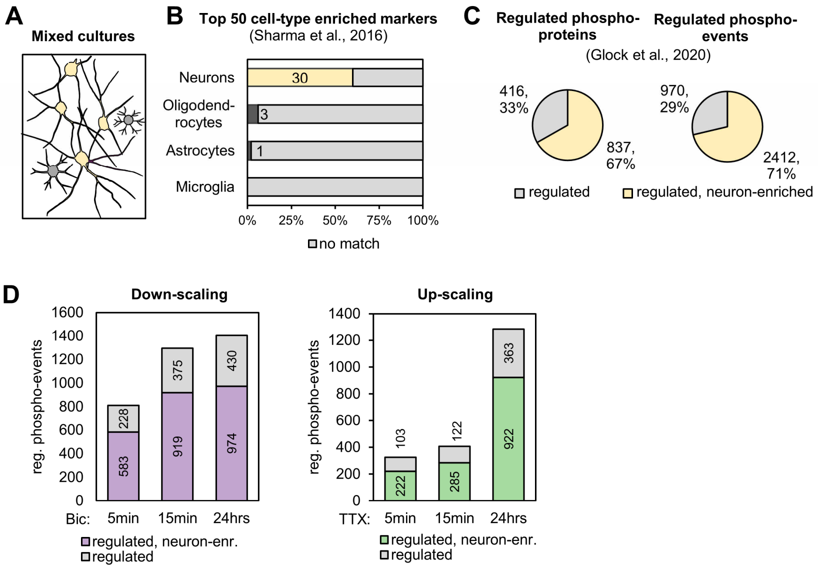

Figure S4 - Excitatory-neuronal contribution to phosphoregulation during homeostatic scaling. Related to Figure 1 and 2.

(A) Scheme representing the mixed primary cortical cell cultures that were used for the phosphoproteomic analysis.

(B) Matching of established, cell-type specific marker proteins to the regulated phosphoproteome. Expression profiles have been previously reported (Sharma et al., 2015) and the top 50 expressed proteins (log_2 fold change compared to all others) for each cell-type were selected and vetted.

(C) Pie chart indicate the proportion of excitatory-neuron specific markers on the level of regulated phosphoproteins (left; 67%) or regulated phospho-events (right; 71%). Markers were matched by gene name. The neuron-enriched fraction is highlighted in yellow. The classifier to bioinformatically filter for transcripts preferentially expressed in excitatory neurons has been previously reported (Glock et al., 2020); for details see Methods.

(D) Bar charts show the number of regulated phosphorylation events per time point during either down-scaling (left) or up-scaling (right) highlighting the proportion of phospho-events on excitatory neuron-enriched proteins (colored; matched by gene name) using the marker genes reported in (C).

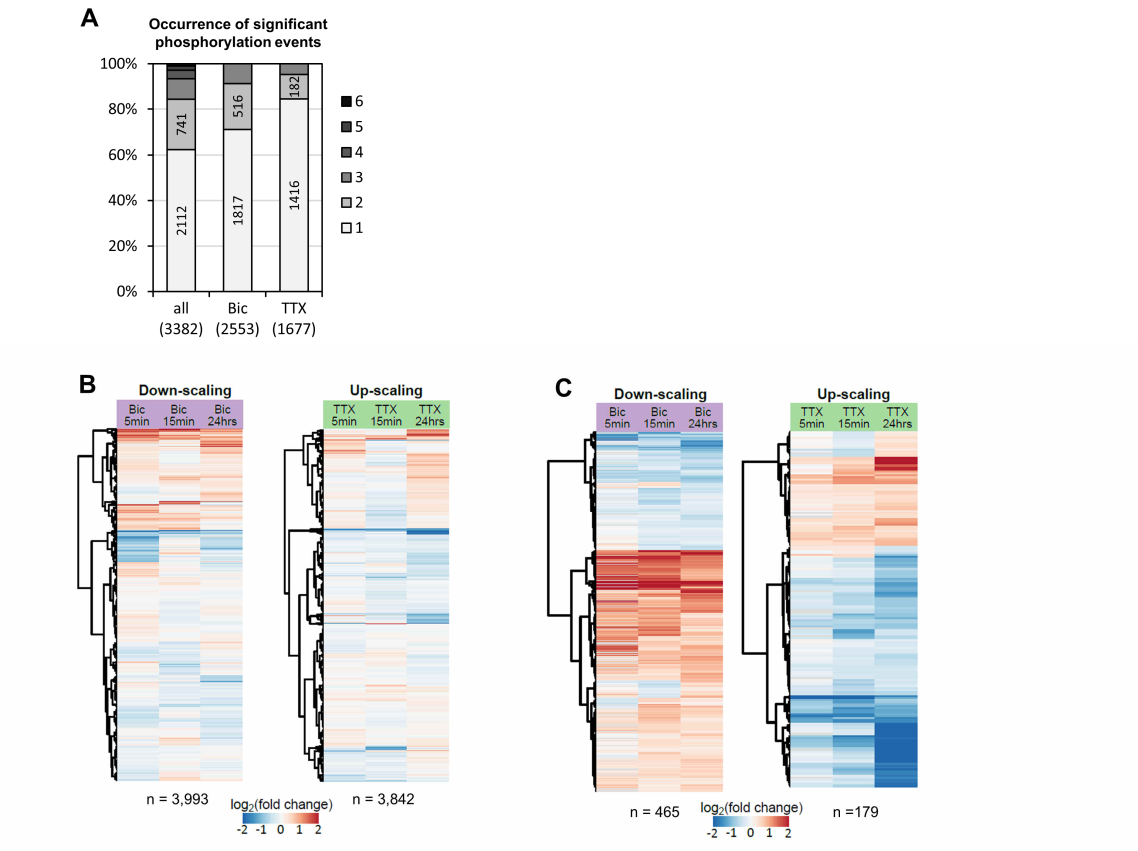

Figure S5 – Significant phosphoregulation across time points. Related to Figure 2 and 3.

(A) Bar chart showing an overview of the occurrence of significantly regulated phosphorylation events across all time points. The first bar summarizes the occurrence of significant regulation of a particular site with respect to all experimental conditions (Bic or TTX: 5 min, 15 min, 24 hrs). 2,112 (62%) of all regulated phosphorylation events were associated with a single time point. The second and third bar summarize the occurrence of significant regulation during up- or down-scaling (n/3). The majority of significant phosphorylation events are regulated in only one of the conditions (down-scaling: 72% and up-scaling 84%)

(B) Temporal profile of the total data landscape of Bic-evoked (left) or TTX-evoked (right) phosphorylation events. Hierarchical clustering (Euclidean distance) was performed on the $\log_2$ fold change of phosphorylation event intensities (treatment vs. control) quantified at each time point of Bic and TTX treatment without applying any filter for significant regulation. The clustering yielded a heatmap with rather diffuse regulation (red: increase in phosphorylation, blue: decrease in phosphorylation), but trending towards persistent regulation which clearly emerged by selection of significant regulators (Figure 2D).

(C) Temporal profile of phosphorylation events significantly regulated at 15 min and 24 hrs (not 5 min) during either Bic- (left) or TTX-stimulation (right). Hierarchical clustering was performed as described in (B). These groups of phospho-events expands the list of bona-fide persistent phospho-events displayed in Figure 2D. Compared to the strictly persistent phospho-events, the number of events in the 'relaxed' categories shown here increases ~2.1 or ~2.6 fold for down- or up-scaling, respectively, while largely maintaining their continuous and switch-like regulatory character.

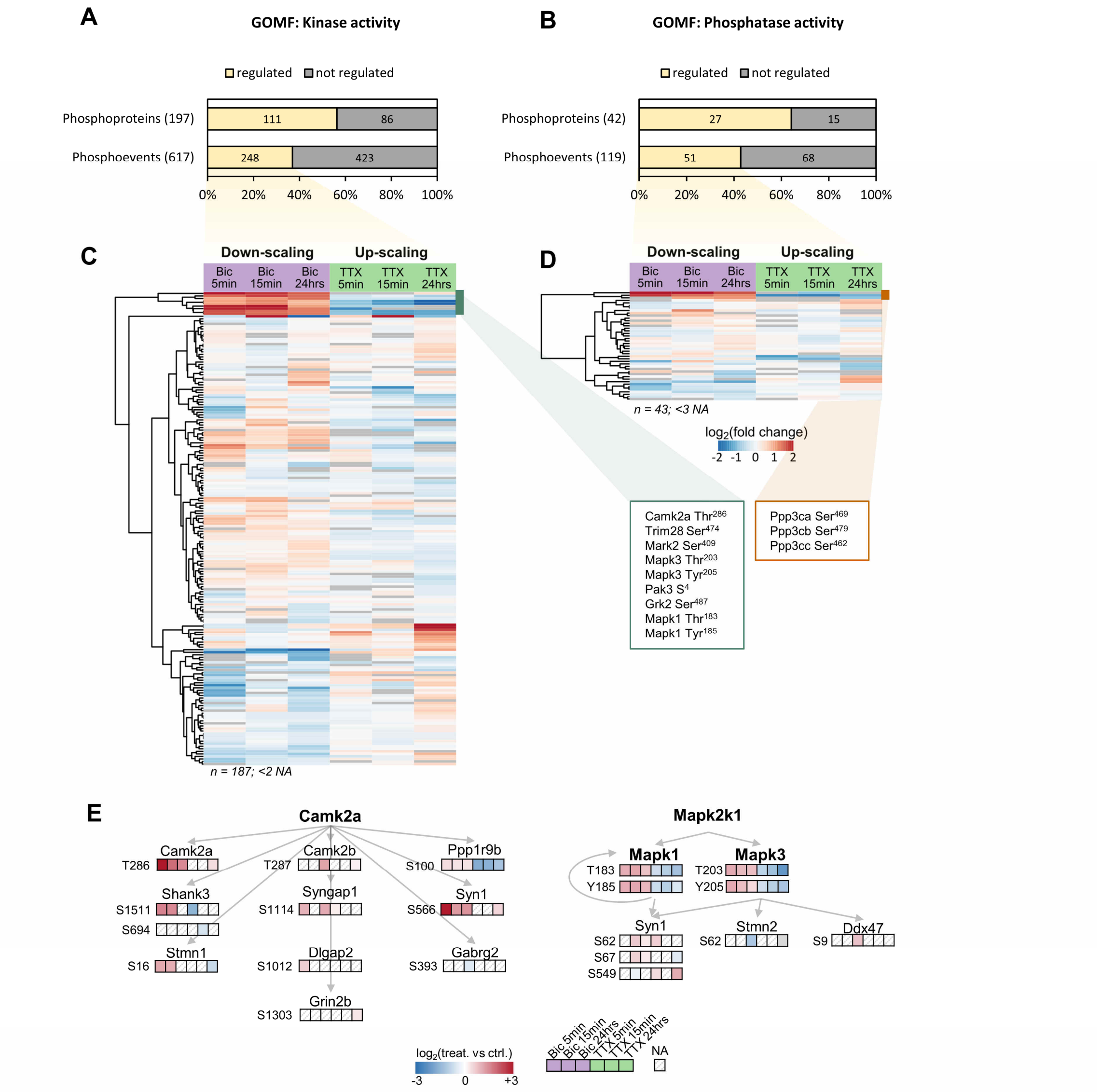

Figure S6 - Phosphoregulated events of kinases and phosphatases. Related to Figure 5.

(A,B) Bar charts indicate the number of all identified kinases (A) or phosphatases (B) on the level of phosphorylated proteins (top row) or phosphorylation events (bottom row). The total number of identifications is indicated in brackets. The proportion of regulated phospho-proteins or -events is highlighted according to significant regulation in at least one time point and condition based on the phosphopeptide-level statistics (FDR < 0.01). Kinases and phosphatases were assigned using the gene ontology annotation for molecular function (GOMF): ‚kinase activity‘ and ‚phosphatase activity‘. We detected phosphoregulation for both, kinases and phosphatases, but identifications and regulatory contribution of phosphatases appeared in a lesser extent compared to the kinases.

(C,D) Heatmap of the $\log_2$-fold changes (intensities of treated vs. control condition) of regulated phosphorylation events that mapped to kinases (C) or phosphatases (D). Phosphorylation events displayed in this heatmaps required a GOMF-term matching kinase or phosphatase activity, significant regulation at at least one condition and were filtered for missing values (NA; grey color) to allow unsupervised clustering (C: < 2 NA; D: < 3 NA). A selection of phospho-sites matching reciprocal clusters were highlighted for both kinases (green box) and phosphatases (orange box).

(E) Phosphorylation status of significantly regulated Camk2a and Mapk1/3 target sites as reported in the rat-specific kinase-substrate database (PhosphoSitePlus) and identified in our dataset. The $\log_2$ fold change of the phosphoregulation (treatment vs. control) is highlighted by color. In case of multiple peptides covering the same phosphorylation site, the $\log_2$ fold change of a particular site was calculated as mean of the events. If the regulation was not significant or if there was no treatment-control pair identified, the box is marked as missing value (NA)

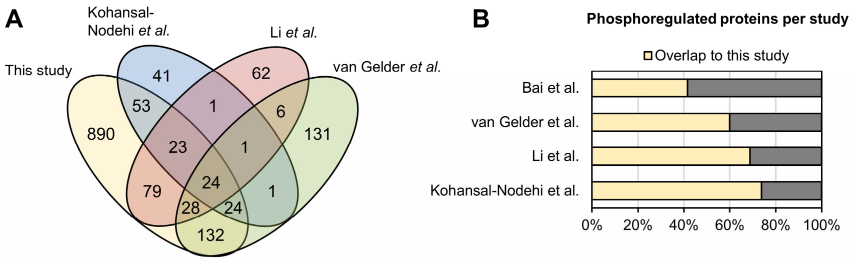

Figure S7 – Comparison of phosphoregulated proteins with other phosphoproteomic studies of synaptic plasticity or neuronal function. Related to Figure 1-5.

(A) Venn diagram highlighting the overlap of proteins that carried at least one significantly regulated phospho-site originating from three different studies focusing on different aspects of synaptic plasticity, such as LTP (Li et al., 2016), depolarization (Kohansal-Nodehi et al., 2016) or mGlur-dependent LTD (van Gelder et al., 2020) with our dataset (for details see Methods).

(B) Barplot indicates the significantly phosphoregulated proteins of each study presented in (A) and including a human AD stage-associated investigation (Bai et al., 2020). Each bar indicates the entity of regulated phosphoproteins per study, the colored fraction highlights the shared phosphoregulated proteins with our dataset (see Methods).
